# Genome-Wide Screening of Broad-Spectrum Resistance to Leaf Rust (*Puccinia triticina* Eriks) in Spring Wheat (*Triticum aestivum* L.)

**DOI:** 10.3389/fpls.2022.921230

**Published:** 2022-06-22

**Authors:** Amira M. I. Mourad, Ibrahim S. Draz, Ghady E. Omar, Andreas Börner, Samar M. Esmail

**Affiliations:** ^1^Leibniz Institute of Plant Genetics and Crop Plant Research (IPK), Gatersleben, Germany; ^2^Department of Agronomy, Faculty of Agriculture, Assiut University, Assiut, Egypt; ^3^Wheat Disease Research Department, Plant Pathology Research Institute, Agricultural Research Center, Giza, Egypt

**Keywords:** leaf rust, seedling resistance, broad spectrum resistance, genome-wide association study, gene models, gene-network

## Abstract

Wheat leaf rust (LR) causes significant yield losses worldwide. In Egypt, resistant cultivars began to lose their efficiency in leaf rust resistance. Therefore, a diverse spring wheat panel was evaluated at the seedling stage to identify new sources of broad-spectrum seedling resistance against the Egyptian *Puccinia triticina* (*Pt*) races. In three different experiments, seedling evaluation was done using *Pt* spores collected from different fields and growing seasons. Highly significant differences were found among experiments confirming the presence of different races population in each experiment. Highly significant differences were found among the tested genotypes confirming the ability to select superior genotypes. Genome-wide association study (GWAS) was conducted for each experiment and a set of 87 markers located within 48 gene models were identified. The identified gene models were associated with disease resistance in wheat. Five gene models were identified to resist all *Pt* races in at least two experiments and could be identified as stable genes under Egyptian conditions. Ten genotypes from five different countries were stable against all the tested *Pt* races but showed different degrees of resistance.

## Highlights

A genome-wide association analysis using different genotyping methods identified different loci for leaf rust broad-spectrum resistance. Resistant diverse genotypes were selected and could be used in future breeding programs.

## Introduction

Wheat (*Triticum aestivum* L.) is one of the major cereal crops in Egypt and all over the world. During its life cycle, wheat suffers from many diseases which reduce its yield and productivity. Leaf rust (LR) caused by *Puccinia triticina* Eriks (*Pt*), is one of the foliar fungal diseases that could cause significant yield losses in epidemic infection cases in the world (Fatima et al., 2020). LR was reported as one of the most widespread and destructive wheat diseases in Egypt (Orabey et al., 2017). As a fungal disease, LR has the adaptation ability to various climate conditions thus, it occurs in many wheat planting areas and causing yield losses which could reach 70% (Kolmer, 2013; Aktar-Uz-Zaman et al., 2017; Rollar et al., 2021). The most effective, environmentally friendly, and low-cost approach to manage LR is growing resistant genotypes.

Generally, wheat resistance to LR has been categorized into two types: race-specific resistance (seedling resistance; also known as all stages resistance), and race non-specific resistance (Like the majority of adult plant resistance genes). Unlike adult plant resistance, seedling resistance could confer a broad-spectrum disease resistance that expresses early in wheat life cycle, mainly seedling growth stage and also expressed at all stages of plant growth. The main defect in this type of resistance is that, due to the race adaptation ability, the disease could overcome the resistant genes and produce new virulent races that cause epidemics situation in single gene wheat cultivars (Folr, 1956; Flor, 1971). This is the recent situation in the Egyptian fields where different *Pt* races among the Egyptian governorates and growing seasons have been reported that resulted in a breakdown of the resistance in some important Egyptian cultivars (Omara et al., 2021). To date, more than 80 leaf rust resistance genes (*Lr*) have been identified and formally cataloged in wheat, most of them confer race specific resistance (McIntosh et al., 1995, 2008, 2013; Gill et al., 2019; Bhavani et al., 2021). However, a limited number of effective race-specific resistance genes are available. Some of these genes were postulated to be present in the Egyptian wheat germplasm but became ineffective in the last few years (Draz et al., 2015, 2021; Atia et al., 2021). Pyramiding of effective *Lr* race-specific resistance genes in one or few genotypes is preferred to obtain broad-spectrum disease resistance (Kolmer et al., 2008). Therefore, looking for new sources of *Lr* resistance genes in different genetic backgrounds will improve Egyptian wheat germplasm by combining different resistance genes. Even though, pyramiding different *Lr* genes in few genotypes based on the phenotypic selection is a challenging process due to the misunderstanding of the genetic bases of the resistance in the selected genotypes. Therefore, it is preferred to use molecular markers in selecting resistant genotypes that provide in-depth understanding of the genetic control of the resistance by using tightly linked markers with different resistance genes.

To identify genomic regions controlling the target traits, genome-wide association study (GWAS) could be used. GWAS was used widely to identify marker trait associations (MTAs) with different traits in wheat including yield and traits correlated with biotic and abiotic stress resistance/tolerance (Wang et al., 2014, 2017; Sallam and Martsch, 2015; Sallam et al., 2017, 2018, 2019; Mourad et al., 2018a,b, 2019a,b; Abou-Zeid and Mourad, 2021; Eltaher et al., 2021b; Rollar et al., 2021; Zhang et al., 2021). It depends mainly on linkage disequilibrium between the phenotypic variation of the studied trait and molecular markers (Alqudaha et al., 2019). However, due to the rapid development in molecular marker techniques, many high-throughput genotyping platforms, such as Diversity Arrays Technology (DArT), iselect arrays including 9K and 90K, and genotyping-by-sequencing (GBS) have been appeared and used widely in wheat for gene discovery and genomic prediction. The presence of such a wide range of genotyping methods confusing plant genome researcher of which method they should use in their research. These methods differ in their costs as well as their accuracy in detecting MTAs (Bajgain et al., 2016; Elbasyoni et al., 2018; Ishikawa et al., 2018; Darrier et al., 2019; Liu et al., 2020).

The objectives of the recent study were to (1) understand the genetic variation in LR resistance in a diverse spring wheat panel consisting of 198 accessions/cultivars that were collected from 22 different countries all over the world and adapted well to the Egyptian field conditions, (2) identify MTAs associated with LR resistance using three different types of genotyping markers sets that are: GBS-SNP markers, iselect 9K-SNP array, and DArT markers, and (3) select the best genotypes that could be used in future breeding programs to improve LR resistance in the Egyptian wheat germplasm.

## Materials and Methods

### Plant Materials and Experimental Design

In the current study, a set of 198 spring wheat genotypes were evaluated for LR resistance at seedling growth stage. These genotypes represent 22 different countries around the world (Supplementary Table S1; Supplementary Figure S1). The seeds of these genotypes were obtained from the USDA-ARS, Aberdeen, ID, United States, except the seeds of the Egyptian cultivars that were obtained from the Egyptian governorate. Most of the tested genotypes (35 genotypes) are Egyptian wheat cultivars that consists of 27 old and new Egyptian cultivars that are planted regularly in the Egyptian fields in addition to eight breeding lines produced by Genetics Department, Faculty of Agriculture, Assuit University, Egypt. The tested genotypes included seven known rust susceptible checks (McNair701, Morocco, Rusty, LMPG-6, Thatcher, Line E, and Little Club; Mashaheet et al., 2020). The remaining 156 genotypes were evaluated under the Egyptian conditions and found to be adapted to these environmental conditions (Mourad et al., 2020; Abou-Zeid and Mourad, 2021). In addition, a total of 65 near isogeneic lines each carry single or multiple *Lr* resistance genes were evaluated for their seedling resistance (Supplementary Table S2). Seeds of these genotypes were obtained from the CIMMYT.

### Evaluation of Leaf Rust Seedling Resistance

Previous studies reported the presence of many *Pt* races that differ by year and location in Egypt (Omara et al., 2021). Therefore, in this study, three different *Pt* bulk spore populations were collected from Alexanderia fields in 2021, Kafrelsheikh fields in 2020, and Kafrelsheikh fields in 2021. The resistance of genotypes was separately tested against each *Pt* population in two greenhouses: (1) Wheat Disease Research Department, Sakha Agricultural Research Station, Agric. Res. Centre, Kafrelsheikh using the spores collected from Kafrelsheikh fields in 2020 (Exp.S.I.) and 2021 (Exp.S.II.), and (2) El-Sabahia Agricultural Research Station, Alexandria using spores collected from Alexandria fields in 2021 (Exp.A.). The experimental design was randomized complete block design (RCBD) with three replications/exp. Each tested genotype was sown in plastic pots (10 cm. diameter)/replication with 10 kernels/pot. The planting was done in clay soils and plants were irrigated as recommended. The *Pt* races of each experiment (Exp.S.I, Exp.S.II, and Exp.A) were identified by Omara et al. (2021) and summarized in Supplementary Table S3.

### Seedling Inoculation With Leaf Rust Bulk Pt Spores

Eight-days-old seedlings were inoculated with the *Pt* urediniospors bulk populations. The inoculation was carried out using Roelfs et al. (1992) method by rubbing the seedling leaves gently between moist fingers with tap water, sprayed in the incubation chambers with water, then inoculated by shaking or brushing the urediniospores over the wheat plant leaves then re-sprayed gently with water in order to induce thin film of free water on the plants which is essential for spore germination and the establishment of infection. The inoculated seedlings were then incubated in a dark dew chamber overnight at 18°C and 95% relative humidity to allow the *P. triticina* spores to germinate and cause infection. The inoculated plants were then moved to the benches in the greenhouse and maintained at 19–22°C and 95–100% relative humidity then kept under observation until the rust pustules are developed. Light intensity was supplied at about 7,600 lux in a photoperiod of 16 h light and 8 h dark (Ohm and Shaner, 1976). Two weeks after inoculation, seedling infection types were scored using 0–4 scale as described by Roelfs et al. (1992).

### Statistical Analysis of Leaf Rust Resistance

To conduct the statistical analyses of seedling response to LR, the ITs obtained based on Roelfs et al. (1992) scale were converted to a linear scale according to Zhang et al. (2014) as described in Rollar et al. (2021). Based on the converted infection type, hereinafter known as linear scale (LIT), immune genotypes are those that had IT 0, 0–2, 2–5, and 5–9, were identified as immune (I), resistant (R), moderately resistant (MR), and susceptible (S) genotypes, respectively. LIT data from the three experiments was combined and ANOVA was conducted using PLABSTAT software (Utz, 1997) based on the following model:


$$Y_{ijk}=\mu +g_{i}+r_{j}+E_{k}+gE_{ik}+e_{ijk}$$

Where, *Y_ijk_* is an observation of genotype *i* in replication *j* which was planted in experiment *k*, *μ* is the general mean; *g_i_*, *r_i_*, and *E_k_* are the main effects of genotypes (fixed effects), replications and experiments (random effects), respectively; *gE_ik_* the performance of each genotype in each experiment, *e_ijk_* is the error.

For each experiment, LIT data from the three replications was combined and used for ANOVA by PLABSTAT software (Utz, 1997) using the following model:


$$Y_{ij}=\mu +r_{j}+g_{i}+gr_{ij}+e_{ij}$$

Where *Y_ij_* is an observation of genotype *i* in replication *j*, *μ* is the general mean; *g_i_*, *r_j_* are the main effects of genotypes and replications, respectively; *e_ij_* is the error. The BLUPs values were calculated following the same model using lme4 R package (Bates et al., 2015). Broad-sense heritability was calculated using the following formula:


$$H^{2}=\sigma_{G}^{2}/\left(\sigma_{G}^{2}+\frac{\sigma_{GR}^{2}}{r}\right)$$

where $\sigma_{G}^{2}$ and $\sigma_{R}^{2}$ are the variance of the lines and the residuals, respectively. *r* is the number of replicates.

### Genotyping of the Tested Diverse Spring Wheat Genotypes and PCA

In the recent study, three different types of genotyping data sets were available for the diverse genotypes as following:

- Genotyping-by-Sequencing (GBS): for this purpose, DNA was extracted from 2 weeks old seedlings of 103 genotypes using BioSprint 96 DNA Plant Kits (Qiagen, Hombrechtikon, Switzerland). The extracted DNA was digested for GBS purpose as described in Mourad et al. (2020). SNP calling was done using Chinese Spring genome assembly from the International Wheat Genome Sequencing Consortium (IWGSC) Reference Sequence v1.0 as the reference genome. SNP markers identified were filtered for maximum missing sites per SNP < 20%, minor allele frequency (MAF > 0.05), and maximum missing sites per genotype <20% (Belamkar et al., 2016; Mourad et al., 2018b, 2019b). After filtration, heterozygous loci were marked as missing, and the SNP markers were re-filtered using the same filtering criteria. The purpose of removing heterozygous loci is to obtain better estimation of allele effect (Mourad et al., 2018a; Abou-Zeid and Mourad, 2021; Supplementary Table S1).
- Wheat iSelect 9K SNP array: Out of the 198 tested genotypes, a set of 156 genotypes have genotyping 9K SNP array data with a number of 6,883 SNPs covering the whole 21 chromosomes and provided by Gao et al. (2017) (Supplementary Table S1).
- Diversity Arrays Technology Marker (DArT): 139 genotypes were genotyped using 437 DArT markers as a part of Maccaferri et al. (2015)’ QTL-mapping study for stripe rust resistance in the United States fields. Marker data is available on the U.S. National Plant Genome system^1^ (Supplementary Table S1).

The principal component analysis (PCA) was done using each set of the available genotyping data set separately using TASSEL 5.0 software (Bradbury et al., 2007).

### GWAS for Leaf Rust Seedling Resistance

For each experiment, BLUPs values were used to conduct genome-wide association study using each set of genotyping data. The BLUPs values were calculated using lme4 R package (Bates et al., 2015). The GWAS analysis was done following three different models: Generalized Linear Model (GLM), Mixed Linear Model (MLM), and Fixed and random model Circulating Probability Unification (FarmCPU) using rMVP R package (Yin et al., 2021). Each model included PCA and/or kinship as a covariate. The best model for each experiment was determined based on the deviation of the distribution of the observed −log10 *p*-value from the expected values in the QQ-plot. The significant SNP markers were identified as those who have *p*-value < 0.001 (−log10 > 3.00). Based on LIT, smaller values represent resistant genotypes and large values represents susceptible genotypes. As a result, target allele of each significant marker was detected as the one that has a lower marker effect. The allelic effect as well as the phenotypic variation explained by marker (*R^2^*) were calculated using TASSEL software (Bradbury et al., 2007). Linkage disequilibrium (*r^2^*) between each pair of the significant SNPs located on the same chromosome was visualized as a heatmap using LDheatmap R package (Shin et al., 2006). Significant genomic regions/markers were visualized using MapChart software (Voorrips, 2002).

### Gene Models Underlying the Significant SNPs, Their Functional Annotations, Gene Network, and Gene Expression

To further investigate and confirm the GWAS results, high confidence gene models harboring significant markers were identified. For each GBS-SNP marker significantly associated with LR resistance, gene models harboring the position of the significant SNPs was detected using *EnsemblePlants* database available on https://plants.ensembl.org/Triticum_aestivum/Info/Index. For 9K SNP array, the sequence of the significant SNPs was obtained from GrainGenes data base^2^ then blasted against the wheat genome using *EnsemblePlants* database. The identified Significant DArT markers also blasted against the wheat genome using the same database after obtaining their sequence from Diversity Arrays Technology Sequences available on https://www.diversityarrays.com/technology-and-resources/sequences/. After blasting the 9K SNP/DArT marker sequence, the position was detected based on the chromosomal location of the marker, the highest length of the blast, the highest percentage of identity (ID%) and the lowest *p*-value as described in (Mourad et al., 2021).

After detecting these high confidence gene models harboring the significant markers, their functional annotation was investigated based on the genome annotations provided by International Wheat Genome Sequence Consortium (IWGSC) v.1.0 and examined for their association with LR resistance. In addition, the relationship between the identified gene models and known disease resistance genes/QTLs was tested and visualized as a network using KnetMiner website.^3^ The expression of the identified genes was compared under disease and controlled conditions using Wheat Expression Browser available on^4^ to provide more understanding of the identified gene models.

## Results

### Evaluation of Leaf Rust Isogenic Lines and Susceptible Checks

To investigate the effective resistant genes to the Egyptian *Pt* races, 45 near isogenic lines carrying 48 different *Lr* resistance genes were included in Exp.S.I. and Exp.S.II. Out of these lines, only one genotype carrying *Lr57* gene was immune against the tested races in both experiments (Supplementary Table S2). Furthermore, *Lr23* and *Lr51* were immune in only one experiment. In addition, 12 resistance genes, *Lr45*, *Lr13*, *Lr10*, *Lr10 + 27 + 31*, *Lr19*, *Lr22a*, *Lr35*, *Lr53*, *Lr47*, *Lr18*, and *Lr25*, were found to be R in one experiment and MR in the second one. The remaining 31 resistant genes were found to be ineffective against the Egyptian races in Exp.S.I. and Exp.S.II as they represented IT ≥2 (LIT≥5). In addition, a set of 20 lines carrying different 20 *Lr* resistance genes were available and evaluated in Exp.A. (Supplementary Table S2). All the tested genes were found to be ineffective against *Pt* races in El-Sabahia with IT >2. These genes were also ineffective in Exp.S.I. and Exp.S.II.

**Figure 1:**
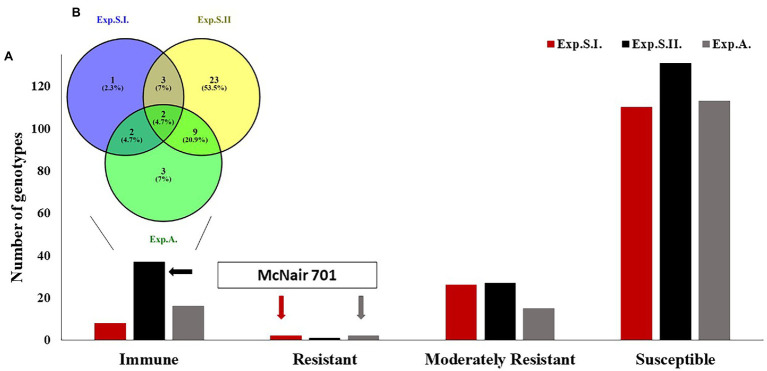
The response of the tested wheat diverse panel to the different leaf races in Exp.S.I., Exp.S.II., and Exp.A. **(A)** The number of immune, resistant, moderately resistant, and susceptible genotypes in each experiment. **(B)** Number of common immune genotypes among the experiments.

To investigate the successes of the artificial infection in the current seedling experiments, seven susceptible checks were included in all the experiments (Supplementary Table S1). Out of the seven susceptible checks, six checks (Rusty, Morocco, LMPG-6, Thatcher, Line E, and Little Club) were highly susceptible against the different tested races with linear IT>8 in the three experiments. While, McNair 701 was immune in Exp.S.II. and resistant in Exp.S.I. and Exp.A. (Figure 1).

### Variation in Seedling Response to Different Races of Leaf Rust in the Tested Spring Wheat Panel

The analysis of variance (ANOVA) reveals highly significant differences among the three conducted experiments (Table 1). In addition, a highly significant genotype × experiment interaction was found. For each experiment, highly significant differences were found among the genotypes with no significant differences between the replications (Supplementary Table S4). High degrees of broad-sense heritability (H^2^) were obtained in the three experiments with a value of 0.98, 0.99, and 0.98 for Exp.S.I., Exp.S.II., and Exp.A., respectively.

**Table 1:** Mean square of leaf rust seedling resistance in 198 spring wheat core collection among the three experiments (Exp.S.I., Exp.S.II., and Exp.A.).

Source of variance	d.f.	Mean square
Experiments (E)	2	432.95^**^
Replications (R)	2	0.34
Genotypes (G)	145	43.00^**^
GE	290	24.76^**^
GR	290	0.24
RE	4	0.37
GER	580	0.26
Total	1,313	–

* *p* < 0.05;

** *p* < 0.01.

Most of the tested genotypes were found to be susceptible (LIT>5) with a number of 110, 131, and 113 genotypes in Exp.S.I., Exp.S.II., and Exp.A., respectively (Figure 1A). While a number of 8, 37, 16 genotypes were immune (LIT = 0) in Exp.S.I., Exp.S.II., and Exp.A, respectively. Furthermore, two, one, and two genotypes were resistant (0 < LIT < 2) in Exp.S.I., Exp.S.II., and Exp.A, respectively. In addition, 26, 27, and 15 genotypes were moderately resistance with LIT values ranging from 2 to 5 in Exp.S.I., Exp.S.II., and Exp.A., respectively (Figure 1A). The number of common resistant genotypes among the three experiments was investigated and presented in Table 2; Figure 1B; Supplementary Figure S2. Out of the tested genotypes, two immune genotypes, Hutch and 15, were found to be common in the three experiments. These genotypes are one from Iran and the other from the United States. Unfortunately, no Egyptian genotypes were found to be immune in all experiments. However, one Egyptian genotype (Misr 2) was found to be common R genotype in all experiments. In addition, seven genotypes were found to be MR to all studied races (Supplementary Figure S2). These MR genotypes were from three different countries as follows: Egypt (four genotypes), Saudi Arabia (two genotypes), and Algeria (one genotype).

**Table 2:** List of common immune, resistant and moderately resistant genotypes in Exp.S.I., Exp.S.II., and Exp.A., their country of origin, and plant ID.

Seedling reaction to leaf rust	Genotype ID	Genotype name	Genotype country	Subpopulation
Immune (IT = 0)	PI_381963	15	Iran	
	PI_595213	Hutch	United States	
Resistant (0 < IT < 2)	–	Misr2	Egypt	
Moderately resistant genotypes (2 < IT < 5)	–	Gimmeiza-12	Egypt	Pop.1
	–	Qadry_001	Egypt	Pop.1
	–	Qadry_006	Egypt	Pop.3
	–	Sohag-5	Egypt	Pop.3
	PI_542675	Sudani	Algeria	Pop.2
	PI_343715	–	Saudi Arabia	Pop.2
	PI_343716	–	Saudi Arabia	

### Association Mapping of Leaf Rust Resistance

#### Genotyping Marker Sets and Principal Component Analysis

The GBS generated a set of 287,798 SNPs for 103 genotypes. After removing the heterozygous cites and quality filtration, a set of 11,362 SNPs was obtained. This set is well distributed across all wheat chromosomes which increases the possibility of identifying marker traits associated (MTA) for LR resistance (Supplementary Figure S3). In addition, the 6,883 SNP markers generated by iselect 9K SNP array and the 437 DArT markers were covering all the 21 wheat chromosomes as well as the ‘unknown’ chromosome. However, marker density for 1 Mb window size/chromosome differs based on the different marker sets which suggesting that each marker set providing different sequencing information (Supplementary Figure S4). Hence, combining all the three sets in the association analysis might provide different results.

Population structure was done previously for the 103 genotypes genotyped by GBS-SNP markers and the presence of three subpopulations was reported (Mourad et al., 2020). In the current study, PCA was done using 9K SNP array (156 genotypes) as well as DArT markers (139 genotypes). The results of PCA using both sets confirm the presence of three subpopulation in the current panel (Supplementary Figure S5). The distribution of the tested genotypes in the different three subpopulations is presented in Supplementary Table S1. Each genotype was located within the same subpopulation based on the three marker data sets except for some genotypes which were located within different subpopulations based on the DArT and 9K-SNP markers compared with the GBS_SNP’ structure results.

#### Association Mapping of Seedling Leaf Rust Resistance in the Three Experiments

**Figure 2:**
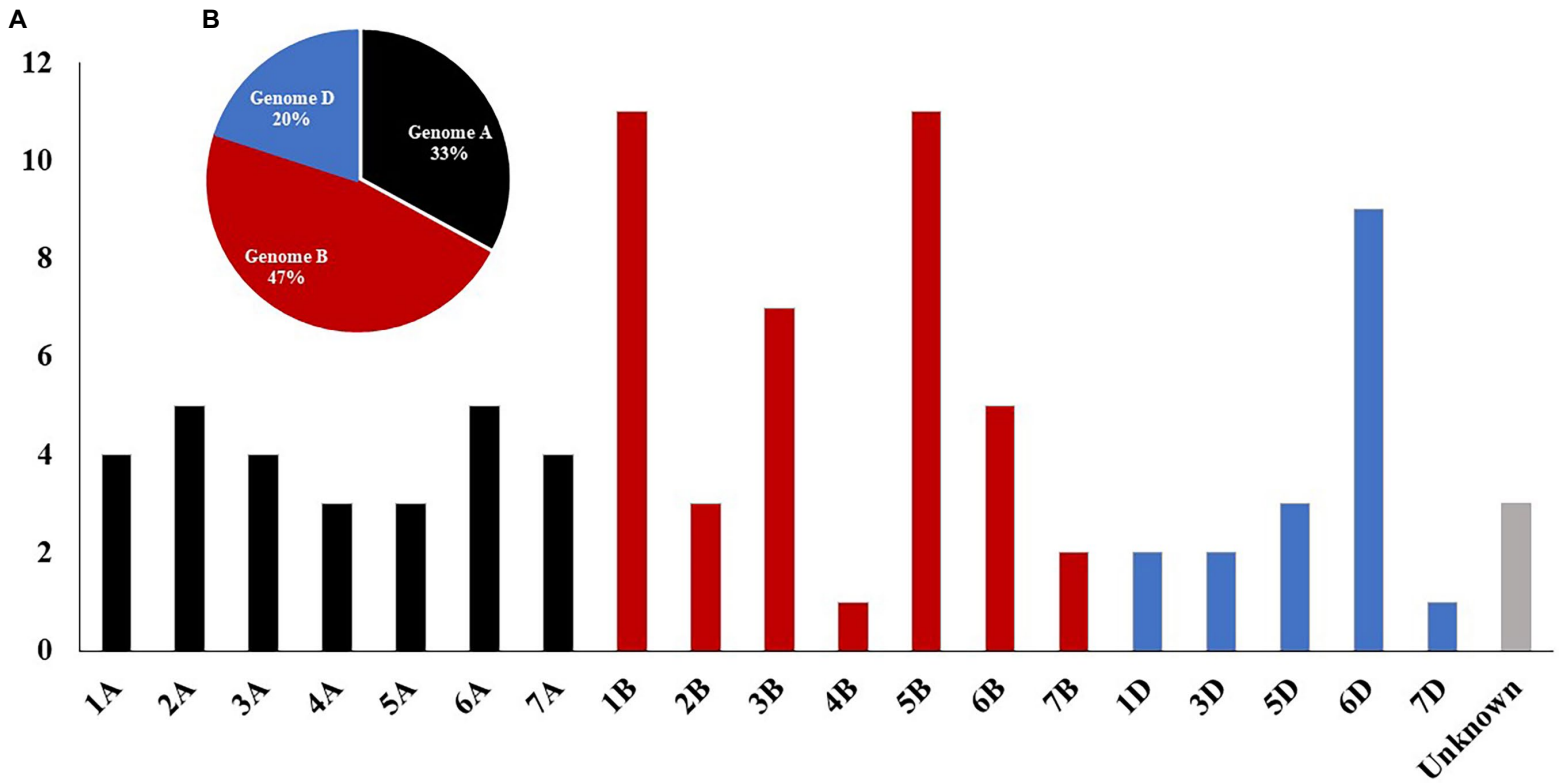
Distribution of total significant markers associated with leaf rust resistance identified by genome-wide association study (GWAS) using GBS-SNP, 9K-SNP array, and DArT markers based on chromosomal location **(A)** and for each genome **(B)**.

Based on the ANOVA results, highly significant differences among the three experiments were found. As a result, GWAS was conducted for each experiment separately. To correct the effect of population structure in the tested panel, principal coordinate (PC) and kinship were included in the GWAS model, and three different models were used for each genotyping data set. A summary of GWAS table with the three marker sets is presented in Table 3, while, the detailed GWAS results is extensively presented in Supplementary Tables S5–S7. A total number of 64, 36, and 7 significant markers was detected using GBS-SNPs, 9K-SNP array, and DArT markers, respectively (Table 3). The total number of significant SNPs detected by the three markers sets in each chromosome is presented in Supplementary Figure S7; Supplementary Tables S5–S7. Chromosomes 1B and 5B were found to be carrying the highest number of significant markers with a number of 11 markers for each chromosome. While both chromosomes 4B and 7D had the lowest number of significant markers with only one marker for each chromosome. No significant markers were located on chromosomes 2D and 4D (Figure 2A). At the genome level, the highest number of the significant SNPs were found in genome B, followed by genome A, and genome D with a percentage of 47, 33, and 20%, respectively (Figure 2B).

**Table 3:** Summary of significant markers identified for each experiment based on genome-wide association study using GBS-SNPs, 9K-SNP array, and DArT markers.

Marker set	Experiment	No. of sign	*R* ^2^	*p*-value	Allele effects	No. of common markers
GBS-SNPs	Exp.S.I.	12	12.20–34.20	1.48E-05 to 0.0008	(−3.01) – (−2.50)	3
	Exp.S.II.	34	6.27–31.376	3.85E-06 to 0.0009	(−4.18) – (−1.91)	
	Exp.A.	18	25.19–44.71	6.57E-06 to 0.0009	(−6.34) – (−2.00)	
9K-SNP array	Exp.S.I.	24	4.76–18.82	9.22E-07 to 0.0009	(−3.71) – (−1.56)	2
	Exp.S.II.	8	3.13–7.48	0.0003 to 0.0009	(−3.03) – (−1.77)	
	Exp.A.	4	5.78–18.64	2.58E-06 to 0.0006	(−3.85) – (−2.25)	
DArT	Exp.S.I.	5	2.51–13.18	4.57E-06 to 0.0009	(−2.20) – (−1.73)	NA
	Exp.S.II.	2	6.05–11.98	0.0006 to 0.0007	(−2.85) – (−1.86)	
	Exp.A.	–	–	–	–	

##### GWAS for Leaf Rust Resistance Using GBS-SNPs Data Set

The highest number of significant markers was identified using this marker data set with a number of 64 GBS-SNP markers were found to be associated with leaf rust resistance in the three experiments (Table 3). The *R*^2^ value of these significant markers ranged from 6.27 to 44.71% and were found to reduce the disease symptoms with a range of 1.91–6.34 degrees.

In Exp. S.I, the result of QQ-plot results representing that FarmCPU+kin model was the most suitable model for detecting marker-trait association (Supplementary Figure S6a). Based on this model, 12 SNP markers were found to be significantly associated with the resistance (*p*-value < 0.001; Supplementary Table S4). These significant SNPs were found to be distributed on four chromosomes (Supplementary Figures S6b, S9). The phenotypic variation explained by theses markers (*R*^2^) ranged from 12.10 to 31.00%. The effect of targeted allele of theses SNPs were found to reduce the symptoms of LR from 2.02 to 3.84 degrees for S6B_439356897 and S3B_221532349 SNPs, respectively.

In Exp.S.II., the best GWAS model was FarmCPU+kin based on the QQ-Plot (Supplementary Figures S6c,d). Based on the results of this model, a set of 34 significant SNPs on 14 different chromosomes were identified (Supplementary Figure S9; Supplementary Table S5). The phenotypic variation explained by theses markers (*R*^2^) ranged from 7.01% for S1B_591225790 marker to 22.48% for S1A_14577853, S1A_14577872, and S1A_14577886 markers, respectively. The effect of the target allele for the significant SNPs ranged from −1.94 to −4.18 for S1B_591225790 and S1A_14577853, S1A_14577872, and S1A_14577886 markers, respectively.

Finally, the GWAS was conducted using the same GBS-SNPs to identify the significant markers controlling the resistance in Exp.A. The best GWAS model which represents the best QQ-plot was GLM + PCA + Kin (Supplementary Figures S6e,f). Based on this model, a set of 18 SNP markers located on seven different chromosomes were detected (*p*-value <0.001; Supplementary Figure S9; Supplementary Table S6). The phenotypic variation of the significant SNPs ranged from 10.61% for marker S3B_60737258 to 44.71% for marker S2A_723067443. The allele effect of these SNPs was found to reduce LR symptoms with a range of −1.99 for S3B_60737258 to −3.50 for both markers S1D_463166242 and S1D_463166267.

**Figure 3:**
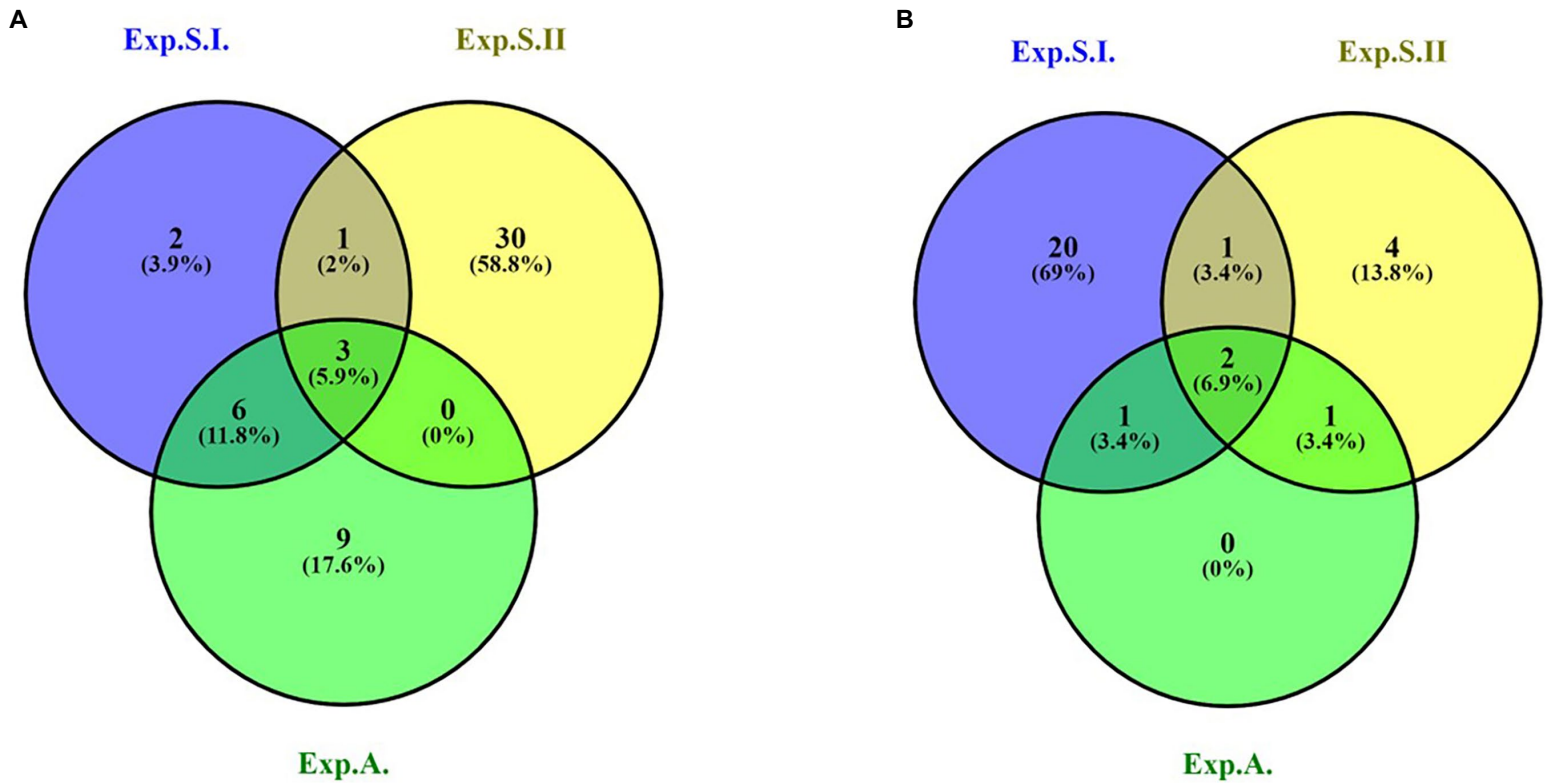
Number of significant GBS-SNP **(A)**, 9K-SNP array **(B)** markers in Exp.S.I., Exp.S.II., and Exp.A and common markers among all the experiments.

Out of all the GBS-SNP markers identified in the three experiments, only three SNPs were found to be associated to leaf rust resistance in the three experiments (Figure 3A; Table 3). These SNPs located on chromosome 6D and had the same *R*^2^ in Exp.S.I., Exp.S.II., and Exp.A. with a value of 31.0, 34.0, and 21.5%, respectively. The target allele of all the three SNPs was found to reduce the LR symptoms with the same degree in Exp.S.I., Exp.S.II., and Exp.A. with a value of 3.01, 3.36, and 2.5 for, respectively (Table 4).

**Table 4:** List of common significant markers in the different sequencing method among the three experiments (Exp.S.I., Exp.S.II., and Exp.A.), their chromosomal location, *p*-value, phenotypic variation explained by marker (*R*^2^), target allele and allele effect.

Marker set	Marker	Chromosome	*p*-value			*R* ^2^ (%)			Target allele	Allele effect		
			Exp.S.I.	Exp.S.II	Exp.A.	Exp.S.I.	Exp.S.II	Exp.A.		Exp.S.I.	Exp.S.II	Exp.A.
GBS-SNP	S6D_464178573	6D	1.48E-05	8.00E-05	0.0003	31.0	18.04	21.54	G	−3.01	−3.36	−2.46
	S6D_464178581		1.48E-05	8.00E-05	0.0003	31.0	18.04	21.54	A	−3.01	−3.36	−2.46
	S6D_464178592		1.48E-05	8.00E-05	0.0003	31.0	18.04	21.54	C	−3.01	−3.36	−2.46
	S6D_465040668^*^		0.00015	0.00034	–	34.20	20.37	–	T	−3.13	−3.50	–
9K-SNP array	wsnp_Ex_c39616_46871127^*^	1B	0.00033	0.00079	–	11.87	6.97	–	G	−1.87	−2.23	–
	wsnp_Ku_c19587_29102203	6A	9.22E-07	0.0004	2.58E-06	18.82	7.47	18.64	G	−3.71	−3.03	−3.85
	wsnp_Ku_c19587_29102203128	6D	9.22E-07	0.0004	2.58E-06	18.82	7.47	18.64	G	−3.71	−3.03	−3.85

* Marker is common between Exp.S.I. and Exp.S.II. and was not significant in Exp.A.

##### GWAS for Leaf Rust Resistance Using 9K SNP Array

Using this marker data set, a total number of 36 markers were found to be associated with leaf rust resistance in the three experiments (Table 3). The *R*^2^ value of these significant markers ranged from 3.13 to 18.82% and were found to reduce the disease symptoms with a range of 1.77–3.85 degrees.

Based on the QQ-plot of the GWAS results, the best model for the 9K SNP array was GLM + PCA + Kinship for Exp.S.I as it represents a perfect distribution of the observed and expected −log10(*p*-value; Supplementary Figures S7a,b). A set of 24 SNP markers distributed on nine different chromosomes were found to be significantly associated with the resistance (*p*-value < 0.001) in this experiment (Supplementary Figure S9; Supplementary Table S4). The phenotypic variation explained by these significant markers ranged from 4.46% for wsnp_Ku_c13043_20902807 marker to 18.82% for both wsnp_Ku_c19587_29102203 and wsnp_Ku_c19587_29102203128 markers. The effect of target alleles of the significant markers ranged from −1.56 for wsnp_Ku_c13043_20902807 marker to −3.08 for wsnp_Ku_c19587_29102203 and wsnp_Ku_c19587_29102203128 markers (Supplementary Table S4).

For Exp.S.II., the best model was GLM + PCA + Kinship which identified eight significant markers on seven different chromosomes (Supplementary Figures S7c,d, S9; Supplementary Table S5). All these markers explained less than 10% of the phenotypic variation (*R*^2^) with a percentage ranged from 3.13% for wsnp_CAP7_c1405_706142 to 7.48% for wsnp_JD_c7305_8404286 marker. The effect of the target alleles reduces LR symptoms with a range of −1.77 for wsnp_CAP7_c1405_706142 marker to −3.03 for both markers wsnp_Ku_c19587_29102203 and wsnp_Ku_c19587_29102203128 (Supplementary Table S5).

Furthermore, a set of four significant markers was identified in Exp.A. based on Kin+FarmCPU model which represented the best distribution of observed and expected −log10(*p*-value; Supplementary Table S6; Supplementary Figures S7e,f). These four markers were located on four different chromosomes and explained a percentage of the phenotypic variation ranged from 5.78 for wsnp_Ex_c28733_37836638 marker to 18.64% for both wsnp_Ku_c19587_29102203 and wsnp_Ku_c19587_29102203128 markers (Supplementary Figure S9). The lowest effect of the target allele was −2.19 for marker wsnp_Ex_c62818_62296518 on chromosome 5B. While the highest allele effect was −3.85 for both wsnp_Ku_c19587_29102203 and wsnp_Ku_c19587_29102203128 markers.

Out of these SNPs, only two markers located on two different chromosomes (6A and 6D) were found to be common in the three experiments (Figure 3B). The phenotypic variation explained by these markers was more than 10% in both Exp.S.I. and Exp.A. While it was lower than 10% in Exp.S.II. (Table 4). The effect of the target allele of each significant SNP was almost the same in all the three experiments (−3.03 < allele effect < −3.85).

##### GWAS for Leaf Rust Resistance Using DArT Markers

The available 437 DArT markers for the tested genotypes were assessed for their association with LR resistance using GWAS. The lowest number of significant markers was identified using this marker set with a number of seven significant markers (Table 3). The *R*^2^ value of these significant markers ranged from 2.51 to 13.18% and were found to reduce the disease symptoms with a range of 1.73–2.85 degrees.

In Exp.S.I, the best GWAS model was Kin+FarmCPU (Supplementary Figures S8a,b). Based on this model, five markers distributed on four chromosomes as follows: 2B (one marker), 3B (two markers), 6B (one marker), and 7B (one marker), were significantly associated with the resistance (*p*-value < 0.001; Supplementary Figure S9; Supplementary Table S4). All the significant markers explained less than 10% of the phenotypic variation of the seedling resistance (*R*^2^) except marker WPT-730892 on chromosome 6B which explained 13.18% of the phenotypic variation. Based on the target allele and its effect, the presence of markers WPT-8412, WPT-0610, and WPT-8615 was associated with increasing the resistance. While the presence of WPT-5672 and WPT-730892 was found to increase the seedling susceptibility to LR.

In Exp.S.II., the best GWAS model was Kin+PCA + FarmCPU (Supplementary Figures S8c,d). Based on this model, only two DArT markers, one on chromosome 1B and the other on chromosome 6B, were found to be significantly associated with LR (*p*-value < 0.001; Supplementary Figure S9; Supplementary Table S5). The WPT-2744 marker located on chromosome 1B was found to explain 12.00% of the phenotypic variation in the tested materials and its presence increases the LR symptoms with 2.85 degree. While WPT-1437 marker on chromosome 6B explained 6.04% of the variation and its presence decreases the symptoms with 1.86 degrees.

The DArT markers were also tested for their association with the resistance in Exp.A. Based on the QQ-plot of the different GWAS models, the best model was PCA + Kin+FarmCPU (Supplementary Figures S8e,f). Using this model, no markers were found to be significantly associated with the resistance using *p*-value < 0.001. Therefore, no significant markers were common among the three experiments.

#### Linkage Disequilibrium Among the Significant Markers Located on the Same Chromosome

The LD (*r*^2^) was calculated among the significant SNPs located on the same chromosome. As it appears from Supplementary Figure S9 which represents all the significant markers identified in Exp.S.I and Exp.S.II. and Exp.A. that GBS-SNP marker, 9K SNP array and DArT markers cover different parts of the wheat genome. However, some markers from the different marker sets located near to each other on the same chromosome. Therefore, LD between the significant markers were investigated to detect if they are controlling the same genomic region or different regions. The LD between each pair of the significant markers is presented in detail in Supplementary Table S7. However, we will focus our results on the LD between the common significant markers among the three experiments as well as the common significant markers between the two experiments conducted in Sakha’s greenhouse (Exp.S.I. and Exp.S.II.). These common markers are located on three different chromosomes (1B, 6A, and 6D).

**Figure 4:**
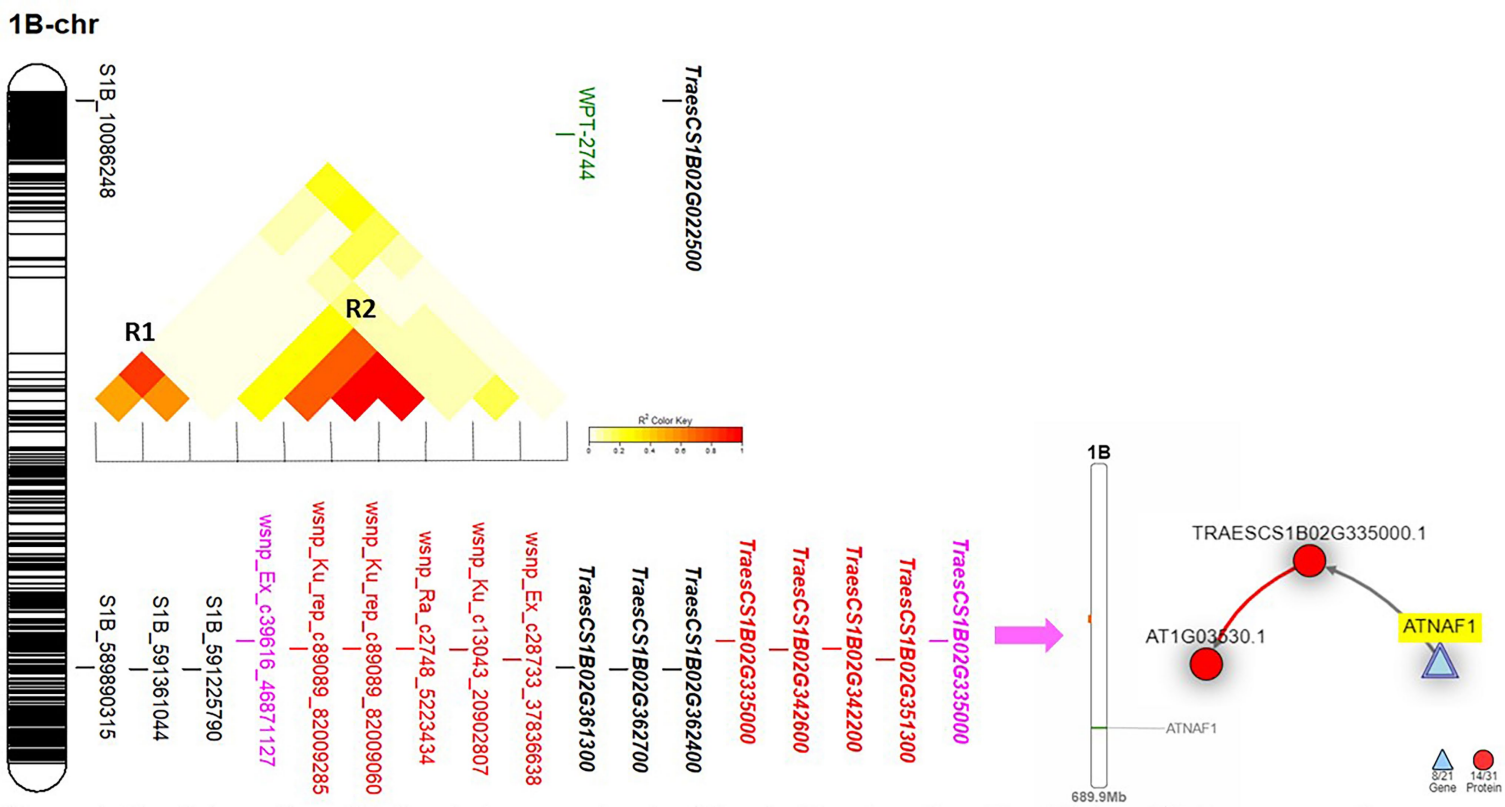
The linkage disequilibrium between each pair of the significant marker identified on 1B chromosome, the gene model they are harboring, and the gene network of the identified gene models in related to disease resistance.

Chromosome 1B was found to carry 11 significant markers in total. Out of these markers, nine markers were located near to each other on the long arm of the chromosome. Only one marker (wsnp_EX_c39616_46871127) was common between Exp.S.I. and Exp.S.II. and located very near to three and five significant GBS-SNP and 9K SNP array markers, respectively (Figure 4). Based on the LD, two genomic regions were identified on this chromosome (R1 and R2). The common significant marker was not located in any of the two identified regions as no significant LD was found between it and the three GBS-SNPs or the remaining five significant 9K SNP array markers.

**Figure 5:**
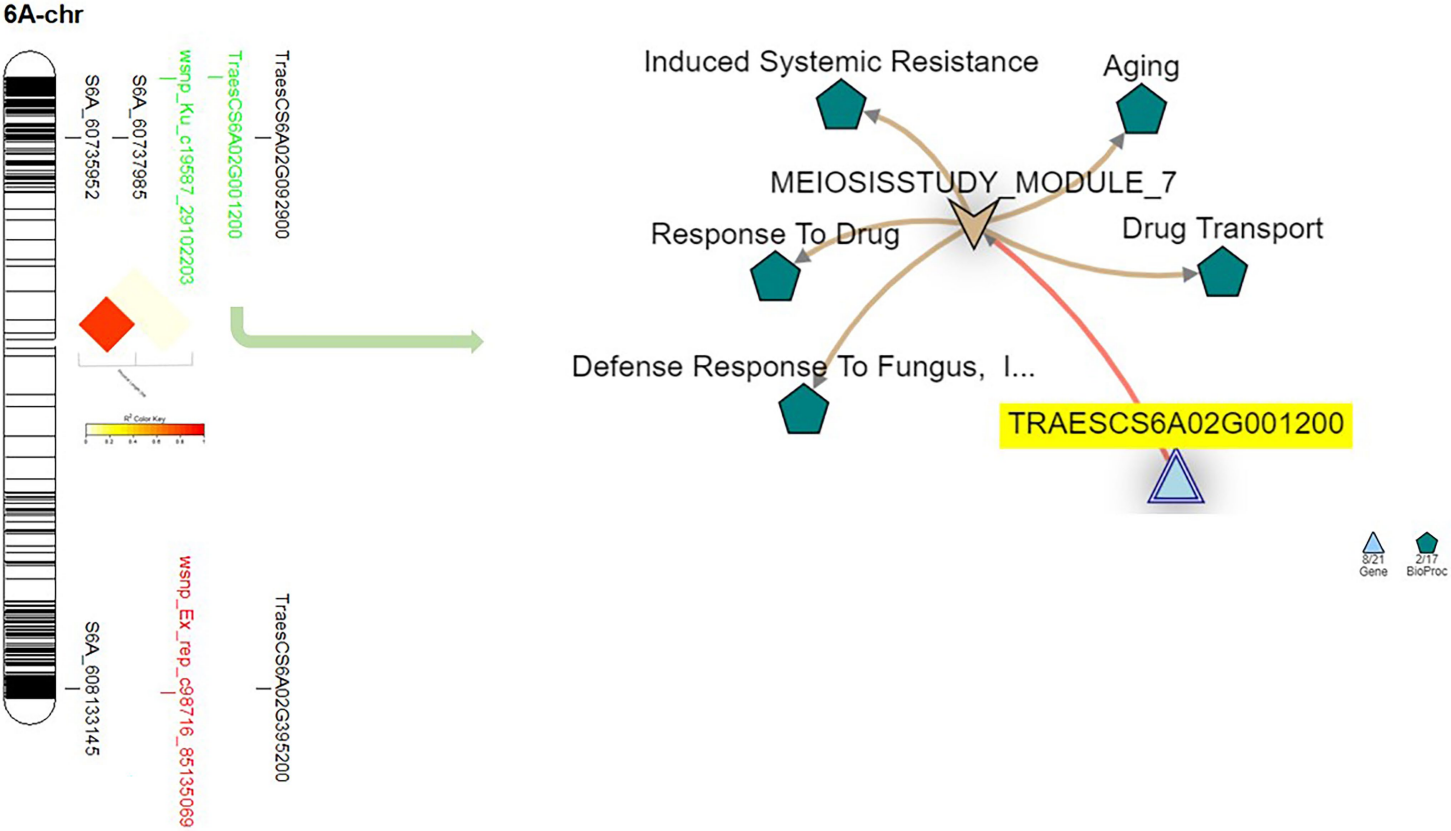
The linkage disequilibrium between each pair of the significant markers identified on 6A chromosome, the gene model they are harboring, and the gene network of the identified gene models in related to disease resistance.

Five significant markers were identified on chromosome 6A (Figure 5). Out of these markers, only one marker (wsnp_ku_c19587_29102203) was common the three experiments. The position of this marker was on the short arm of the chromosome and far a bit from the two significant GBS-SNPs located on the same arm. Therefore, no significant LD was found between this marker and the two GBS-SNPs located near it (Figure 5).

**Figure 6:**
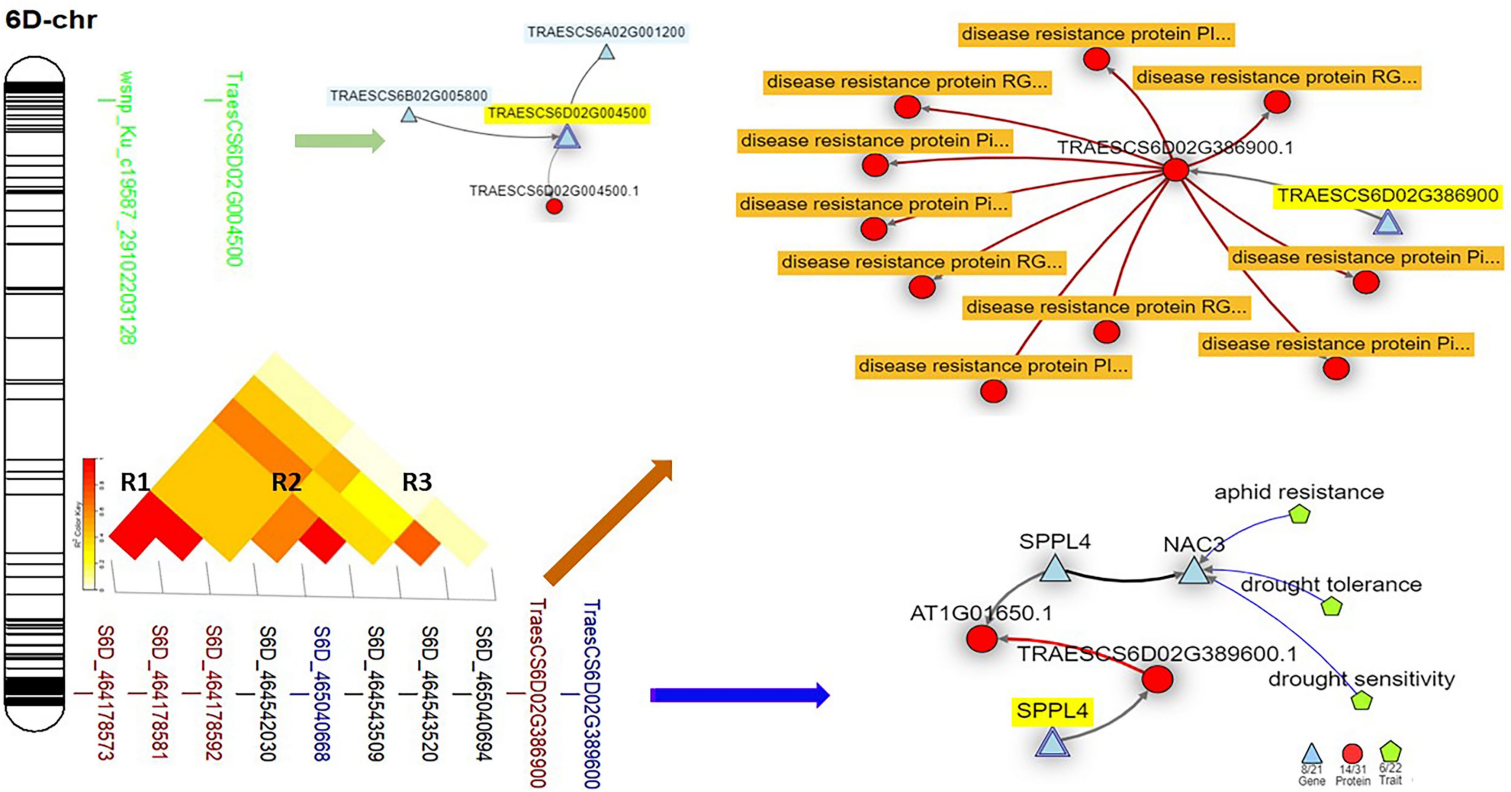
The linkage disequilibrium between each pair of the significant markers identified on 6D chromosome, the gene model they are harboring, and the gene network of the identified gene models in related to disease resistance.

On chromosome 6D, three common GBS-SNP (S6D_464178573, S6D_464178581, S6D_464178592) and one common 9K-SNP array marker (wsnp_Ku_c19587_29102203128) were identified among the three experiments. The sequence and position of wsnp_Ku_c19587_29102203128 marker mapped it on the short arm of the chromosome and far away from the remaining common GBS-SNPs. Based on the LD between the GBS-SNPs, three different genomic regions were identified (R1, R2, and R3). All the three common SNPs are constituting R1 (Figure 6). In addition, one more GBS-SNP marker was common between Exp.S.I. and Exp.S.II. (S6D_464542030). This marker was found to be in high LD with S6D_464542030 marker significant in Exp.S.II. only. Both markers are constituting R2 genomic region.

#### Gene Models Harboring the Significant Markers, Their Functional Annotation, Gene Network, and Gene Expression

To further understand the genetic association of the significant markers identified by GWAS, the gene models harboring these markers were investigated. Furthermore, the functional annotation of the identified gene models and their relationship with the resistance were investigated. In general, a set of 48 gene models was found to harboring the significant markers. The position of these models and their functional annotation is presented in Supplementary Tables S4–S6. In addition, the gene network of these gene models in relation to disease resistance was investigated and presented in Supplementary Figures S10–S13. Most of the gene models were found to have a relationship with stem rust, stripe rust, leaf rust and powdery mildew resistance in wheat. Looking for common gene models identified based on the different three marker sets, no common gene model was found (Supplementary Figure S14). Due to the large number of the identified gene models, we will focus our presentation on the common gene models among the three experiments and common gene models between Exp.S.I. and Exp.S.II. (Table 5).

**Table 5:** List of gene models harboring the common significant markers in Exp.S.I. and Exp.S.II. and their functional annotation.

Sequencing method	Marker	Chromosome	Maker length	Alignment length	Gene model	Gene model position	ID%	Functional annotation
GBS-SNP	S6D_464178573	6D	–	–	TraesCS6D02G386900	464173108-464177628	–	Disease resistance protein RPM1
	S6D_464178581		–	–			–	
	S6D_464178592		–	–			–	
	S6D_465040668^*^		–	–	TraesCS6D02G389600^*^	465040160-465046144	–	Signal peptide peptidase-like protein^*^
9K-SNP array	wsnp_Ex_c39616_46871127^*^	1B	121	121	TraesCS1B02G335000^*^	562374379-562378608	99.3	H/ACA ribonucleoprotein complex non-core subunit NAF
	wsnp_Ku_c19587_29102203	6A	201	65	TraesCS6A02G001200	734214-735473	90.8	Zinc finger protein 512B family
	wsnp_Ku_c19587_29102203128	6D	201	201	TraesCS6D02G004500	2167001-2167940	99.5	Cell surface glycoprotein 1

* Marker and gene model harboring the marker are common between Exp.S.I. and Exp.S.II. and was not significant in Exp.A.

**Figure 7:**
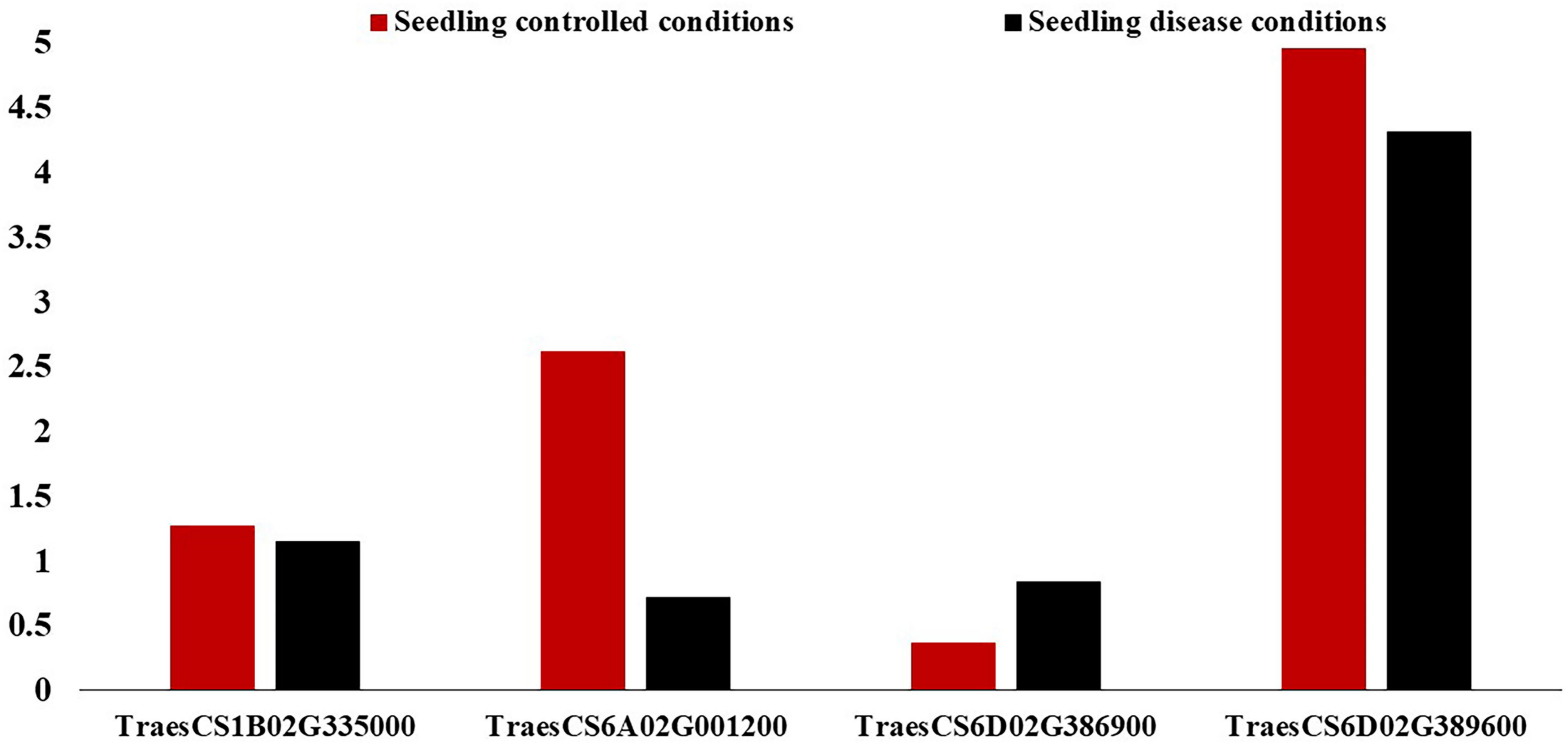
Expression of the identified gene models associated with leaf rust resistance in the leaves/shoots of the tested genotypes in the seedling (red columns) and vegetative (black columns) growth stages under controlled and disease conditions.

On chromosome 1B, one gene model (TraesCS1B02G335000) was found to be common between the Exp.S.I. and Exp.S.II. (Figure 4). The functional annotation of this gene was found to be controlling H/ACA ribonucleoprotein complex non-core subunit NAF (Table 5). There is no information available about the relation between this gene model and disease resistance in wheat based on the gene network. Comparing between the expression of this gene, a small reduction was found under disease conditions compared with controlled conditions (Figure 7).

Chromosome 6A was carrying one common gene model (TraesCS6A02G001200) among the three experiments. The functional annotation of the TraesCS6A02G001200 gene models was found to control zinc finger protein (Table 5). Based on the network of this gene, it was found to have a relation with wheat defense response to fungus and inducing systemic resistance (Figure 5). A huge reduction in the expression of this gene in the seedling leaves was reported under disease conditions compared with controlled conditions (Figure 7).

One gene model (TraesCS6D02G386900) was found to be common among the three experiments on chromosome 6D. In addition, one gene model (TraesCS6D02G389600) was found to be common between Exp.S.I. and Exp.S.II. The functional annotation of the gene model TraesCS6D02G386900 was found to produce PRM1 Disease resistance protein and its network was completely related to disease resistance in wheat (Table 5; Figure 6). The expression of this gene model was found to be duplicated under disease conditions compared with the controlled conditions in the leaves of the infected seedling (Figure 7). TraesCS6D02G389600 gene model was annotated to control the production of signal peptide peptidase-like protein (Table 5). Its gene network represents the relation between this gene and drought tolerance as well as aphid resistance in wheat (Figure 6). A reduction in this gene expression was reported under disease infection compared with controlled conditions (Figure 6).

### Selection of the Superior Genotypes for Leaf Rust Seedling Resistance

**Figure 8:**
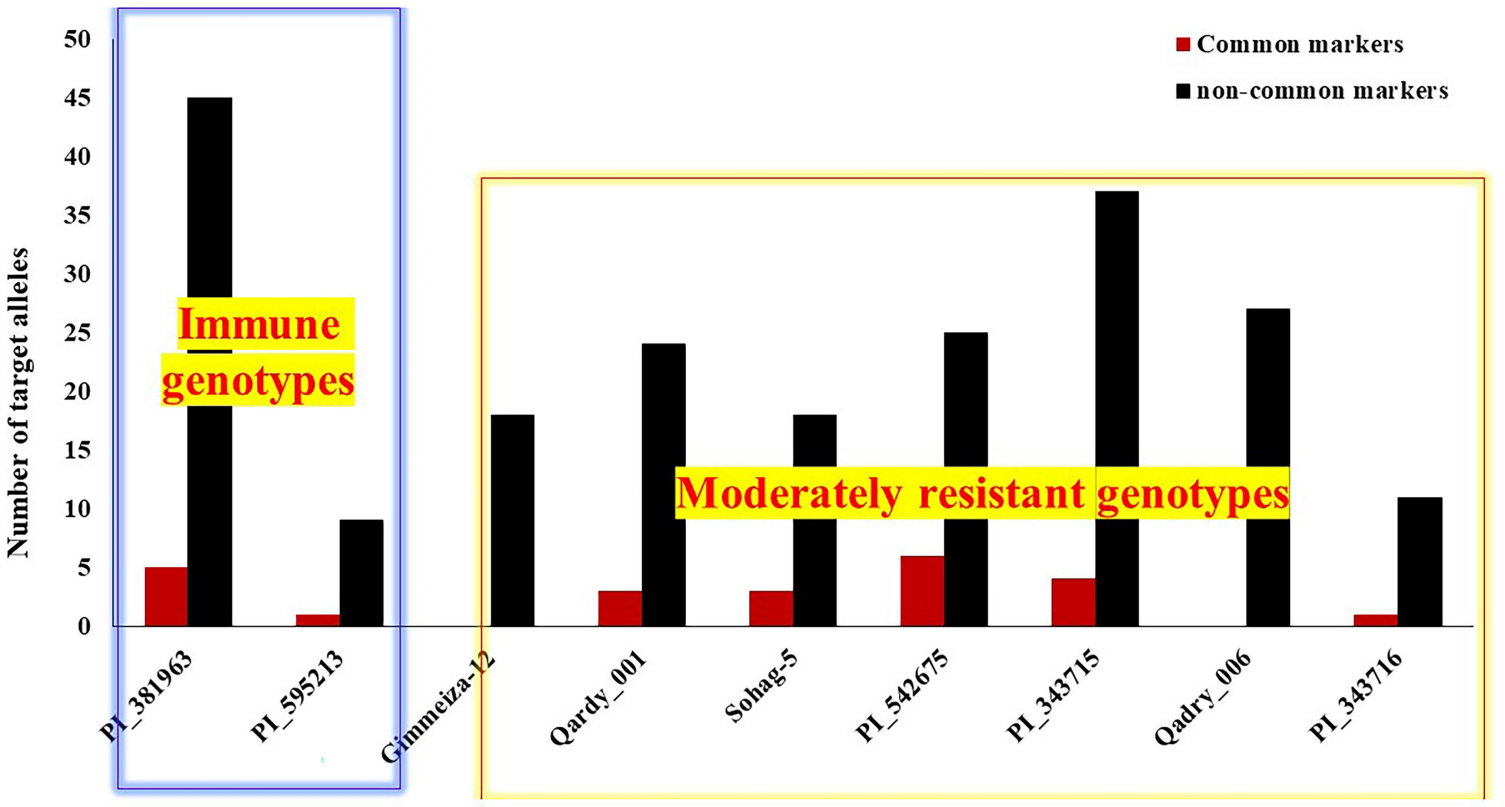
Number of targeted alleles of the significant markers in the two immune and the seven moderately resistant genotypes.

To genetically confirm and understand the resistance in the common resistant genotypes represented in Table 2, the number of targeted alleles, associated with increased resistance to leaf rust, of each common and non-common significant marker was investigated in the immune, resistant, and moderately resistant genotypes. Unfortunately, no marker data were available for the genotype Misr2, which was resistant in all trials (Misr 2). Therefore, the number of targeted alleles presented in it could not be investigated. The number of targeted alleles of the common markers ranged from zero alleles for the two Egyptian genotypes Gimmeiza_12 and Qadry_006 to six alleles in the Algerian genotype (PI_541675). The two immune genotypes had number of one and five targeted alleles of the common markers (Figure 8). For the noncommon markers, the number of targeted alleles ranged from nine to 45 alleles in the American genotype (PI_595213) and the Iranian genotype (PI_381963), respectively. The Egyptian moderately resistant genotypes had an intermediate number of targeted alleles of the noncommon markers.

Furthermore, looking for the subpopulation where the selected genotypes are coming from, we found that the Egyptian MR genotypes were in subpopulations 2 and 3, whereas the immune genotype PI_381963 was in subpopulation 1 based on the STRUCTURE results obtained by the GBS-SNP data set (Supplementary Figure S5). Unfortunately, there was not any available GBS-SNP data for the other immune genotype (Hutch). However, based on the PC analysis using the 9K-SNP array and DArT markers, this genotype was located in subpopulation 1 and subpopulation 2, respectively.

## Discussion

Leaf rust caused by *P. triticina* is one of the most serious diseases that affect the production of wheat in Egypt and all over the world. Recent studies reported the presence of new aggressive *Pt* races that overcame the resistance in the Egyptian cultivars. Therefore, it is urgently needed to produce cultivars with broad-spectrum resistance that can resist as much *Pt* races as possible. Omara et al. (2021) collected *Pt* spores from four different governorates across Egypt including Alexandria and Kafrelsheikh in two growing seasons (2019/2020 and 2020/2021). They found the *Pt* popualtions differ by location and by year within each location (Supplementary Table S3). In the recent study, the same *Pt* populations were used to evaluate a diverse spring wheat panel containing accessions from 22 different countries including Egypt to understand the possibility of improving LR resistance and obtaining genotypes with broad spectrum resistance to LR. This spring wheat panel was found to be genetically highly diverse (Mourad et al., 2020). Understanding the genetic response of such a diverse panel under the Egyptian conditions will help wheat breeders to improve LR resistance in the Egyptian wheat germplasm. The evaluation was done in two important governorates, Kafrelaheikh and Alexandria, that could be considered as sources of LR infection in Egypt. The evaluation was done using *Pt* spores collected from two different growing seasons in Kafrelsheikh (2020 and 2021) and one season in Alexandria (2021) to present a diverse panel of *Pt* races that exists naturally in the fields of these regions. Based on the evaluation of seven LR susceptible checks in both experiments, the recent evaluation could be considered as a valid one as all the checks had LIT ranged from 5 to 9, except McNair 701 which had LIT = 2. Recent studies reported the presence of *Lr25* resistance gene in McNair 701 genotype (Heurta-Espino et al., 2008). This gene was found to be effective against the Egyptian LR races which explain the resistant response of this check in the recent study (Atia et al., 2021). Based on these results, we can conclude that the recent evaluation is a valid one.

### Effective Leaf Rust Resistance Genes Against the Egyptian Races

The evaluation of the LR near isogenic lines was done in each experiment separately to investigate the changes in the genetic control of LR resistance under the Egyptian conditions. Based on the 45 near isogenic lines evaluated in Exp.S.I. and Exp.S.II., only 14 genes are still effective against the Egyptian races. These genes have different response against the *Pt* spores collected from the two different growing seasons which confirms the presence of different races among the different growing seasons. In addition, previous studies reported the effectiveness of *Lr13*, *Lr22a*, *Lr34*, *Lr37*, and *Lr67* genes under the Egyptian conditions (Atia et al., 2021). However, in our recent study, *Lr37*, *Lr34* showed a susceptible reaction with LIT = 8 and 9 for *Lr37* and LIT = 5 and 8 for *Lr34* in Exp.S.I. and Exp.S.II., respectively. Furthermore, nine *Lr* resistance genes (*Lr9*, *Lr10*, *Lr11*, *Lr16*, *Lr18*, *Lr19*, *Lr26*, *Lr27*, *Lr29*, *Lr30*, *Lr34*, *Lr42*, and *Lr46*) were reported previously to produce seedling resistance under the Egyptian conditions (Draz et al., 2015). Based on the results of Exp.A., out of these effective genes, eight genes became ineffective against the Egyptian races as they showed high LIT (LIT = 8 or 9). The results of the isogenic lines evaluated in the three experiments confirmed that the genetic control of LR under Egyptian conditions has been changed and breeding of broad-spectrum resistant genotypes is urgently needed to overcome the effect of such a serious disease under the Egyptian conditions.

### Genetic Variation in Seedling Leaf Rust Resistance in Spring Wheat Diversity Panel

Highly significant differences were found among the three experiments concluded the significant differences in *Pt* races used in each experiment (Table 1). These significant differences were expected as they were inoculated to three different *Pt* populations according to Omara et al. (2021). In addition, the highly significant differences in the tested genotypes in each experiment concluded the high genetic variation in the tested panel which can be used to discriminate the different degrees of leaf rust resistance in the current wheat population. Therefore, selection of the most promising leaf rust resistant genotypes is feasible and can be utilized for future breeding program. The high degree of broad sense heritability indicates that the observed phenotypic variation in all experiments is mainly because genetic variation and the response of the genotypes is stable. Therefore, selection of stable highly resistant genotypes could be done in this experiment. Similar degree of broad-sense heritability was reported previously for LR seedling resistance (Gao et al., 2016; Beukert et al., 2021). Such high degree of broad-sense heritability promised with a genetic improvement for leaf rust resistance against a wide range of *Pt* populations.

The phenotypic response of the tested genotypes ranged from immune to susceptible in all experiments which confirm the presence of genetic variation for LR seedling resistance in the tested panel. Different numbers of immune, R and MR genotypes were found in each experiment. Number of common resistant genotypes from different countries among the three experiments were found. Furthermore, looking for the response of the Egyptian genotypes, no immune genotypes were found in the three experiments except the breeding line Qadry_002 that was immune in Exp.S.I. and Exp.A. and MR in Exp.S.II. Also, four genotypes (Gimmiza-12, Qadry_001, Qadry_006, and Sohag-5) were found to be MR in all experiments (Table 2). Draz et al. (2015) reported the presence of LR seedling resistance in some Egyptian genotypes including Misr2 which confirms our results. However, in their study, many other resistant genotypes such as Sakha94, Giza168, Gemmiza9, Gemmiza10, Gemmiza11, Sids12, Sids13, and Misr1 were found. In our recent study, the resistant genotypes reported by Draz et al. (2015) were found to be highly susceptible (5 < LIT < 9) suggesting that the resistance of these genotypes has been broken and looking for new resistance sources to improve the Egyptian germplasm is urgently needed (Supplementary Table S1). In the recent study, the presence of resistant genotypes from different countries confirms that obtaining broad-spectrum resistance against different Egyptian *Pt* races is possible using this panel due to the presence of immune genotypes from different genetic backgrounds.

### Association Mapping for Leaf Rust Seedling Resistance

#### Accuracy of the Different Marker Sets

Due to the continuous advances in genotyping and marker generating methods, many approaches are available which confuses researchers about the best methods and markers to use in association and genomic predication methods. In our recent study, we used three different marker sets in our GWAS: GBS-SNPs, 9K-SNP array, and DArT markers. Recent research compared the efficiency of these different genotyping method in some crops (Elbasyoni et al., 2018; Ishikawa et al., 2018; Darrier et al., 2019; Liu et al., 2020). However, no previous research included all the three genotyping and marker sets used in the recent study together. The density of the three marker sets per 1 MB window of the different chromosomes suggested that these sets cover different parts of the genome (Supplementary Figure S4). Therefore, combining the three marker sets provides more coverage of the wheat genome. In addition, PCA analysis using each set of the three marker sets suggested the presence of three sub-groups in the studied population despite that some differences in the distribution of the genotypes among the three subpopulations were found based on the three different marker sets (Supplementary Figure S5). However, Liu et al. (2020) concluded that the i-select 9K SNPs array provides less efficient population structure results compared with DArT markers. Furthermore, Heslot et al. (2013) concluded that DArT markers overestimate the genetic diversity compared with GBS data even if the same number of markers/set was used. Hence, GBS markers provide more accurate genetic diversity and population structure analysis. In our study, the largest number of markers was found in the GBS-SNP marker set (11,362 SNPs). Due to the high number of markers in this set and the high accuracy of population structure and genetic diversity, we concluded the presence of three sub-populations in the studied diverse spring wheat panel and trust the distribution of the genotypes among the subpopulations that identified by GBS-SNP markers.

#### GWAS for Seedling Resistance to Egyptian Leaf Rust Races

The high genetic variation and high heritability estimates were very good indicators for identifying alleles associated with target alleles. Moreover, the high genetic diversity among genotypes facilitated the GWAS studies as plant material collected from different countries. The current population was very useful to identify alleles associated with target traits in earlier studies such as drought tolerance (Ahmed et al., 2021), salt tolerance (Hasseb et al., 2022), and stripe rust resistance (Abou-Zeid and Mourad, 2021; Mourad et al., 2021). The number of genotypes in each marker set was suitable for GWAS analysis. At least 100–500 individuals are needed to identify markers associated with target traits in genome-wide association (Kumar et al., 2011; Sallam and Martsch, 2015; Alqudaha et al., 2019).

In the recent study, we used three different models, GLM, MLM, and FarmCPU, which include PCA, kinship, or both of them to correct the effect of population structure and avoid false associations for each marker data set. Out of these models, FarmCPU was found as the best one for all the experiments except for Exp.S.I and Exp.S.II using 9K-SNP array where an over estimation was found. Recent studies reported FarmCPU as an accurate model which identify MTAs for different traits and avoid negative false results obtained by MLM model (Liu et al., 2016; Kaler et al., 2020; Muhammad et al., 2021). Our results concluded the efficiency of FarmCPU model comparing with MLM model. However, due to the overestimation of FarmCPU model in two of our experiments, we can conclude that testing multi models in GWAS analysis is very important to detect the best model. GLM + PC + Kinship model was found as a good model which corrects the overestimation of FarmCPU and MLM models in our study and previous studies (Turuspekov et al., 2016; Abou-Zeid and Mourad, 2021).

Combining the results of the three experiments and the three marker data sets, many MTAs were identified to control LR seedling resistance under the Egyptian conditions. Out of the 22 wheat chromosomes (21 chromosomes and one unknown one), 20 chromosomes were found to carry genomic regions associated with the resistance (Supplementary Figure S9). These regions were found to be harboring by 48 gene models. As LR is considered as a serious problem affecting wheat planting in Egypt long time ago, continuous mutations in the fungus and the plant to overcome each other were happened. Furthermore, many studies have been done to improve wheat resistance against LR under Egyptian conditions (Imbaby et al., 2014; Draz et al., 2015, 2021; Atia et al., 2021; Draz and Abd El-Kreem, 2021). Therefore, the presence of many MTAs across spring wheat genome is not a surprising result. The presence of QTLs controlling LR across the 21 wheat chromosomes was also reported in previous studies (Da Silva et al., 2018; Fatima et al., 2020).

On chromosomes 1B, there was one marker (wsnp_Ex_c39616_46871127) located within TraesCS1B02G335000 gene model common between Exp.S.I. and Exp.S.II. Despite that there was no relation between the functional annotation of this gene and disease resistance based on the gene network, this gene model was found to control the production of ribonucleoprotein complex non-core subunit NAF that was reported to play an indirect important role in protecting wheat seedling against stripe rust (*Pst*) infection (Zhang et al., 2019). NAF is one of the RNA binding proteins that are required for H/ACA box maturation and ribosome biogenesis hence improving cell growth and plant development. Due to the importance of the ribonucleoprotein complex non-core subunit NAF in plant growth, lower expression of its controlling genes under infection conditions is expected. Therefore, lower expression of gene TraesCS1B02G335000 was noticed in the present study (Figure 7). The position of this common marker (562 Mbp) was quite close to previous MTA controlling seedling resistance against multi races of *Pt* in spring wheat (551 Mbp) which confirm our findings (Kumar et al., 2020). Many *Lr* resistance genes were mapped on 1B chromosome such as *Lr26* (Zhang et al., 2021), *Lr33* (Dyck et al., 1987), *Lr44* (Singh et al., 2013), *Lr46* (Singh et al., 2013), *Lr51* (Helguera et al., 2005), *Lr71* (Singh et al., 2013), and *Lr75* (Singla et al., 2017). The near isogenic lines of four genes (*Lr26*, *Lr33*, *Lr44*, and *Lr51*) were available and confirmed the efficiency of only *Lr51* gene against the *Pt* races in Exp.S.I. and Exp.S.II. with ITs of 0 and 2, respectively. The presence of *Lr46* gene in the Sakha-94 Egyptian wheat cultivar was reported previously (Draz and Abd El-Kreem, 2021). However, due to the highly susceptible reaction of this genotype in Exp.S.I. and Exp.S.II. (LIT 8 and 9, respectively) we concluded that this gene is not effective against the Egyptian races present in these experiments, hence the identified markers on this chromosomes are not linked to this gene. Due to the lack of *Lr71* and *Lr75* lines, we could not confirm their efficiency under the Egyptian conditions. Based on our results and previous studies, we concluded that the common marker on this chromosome might be linked to *Lr51* or other genes. More studies are needed to confirm the association between this marker and *Lr51* gene.

On chromosome 6A, one marker with a major effect (wsnp_Ku_c19587_29102203, *R*^2^ = 18.82) was found to be common between Exp.S.I. and Exp.S.II. The gene network of TraesCS6A02G001200 gene model harboring this common marker presents the relationship between this gene and defense response to fungus which confirms our results (Figure 5). Furthermore, this gene was found to functionally controlling the production of cell surface glycoprotein 1, an elicitor which was found to play an important role in fungal resistance in wheat (Takahashi et al., 2006). Previous studies reported that glycosylated proteins act as a barriers in plant defense against pathogens (Lin et al., 2020). However, the synthesis of this protein in infected plants was noticed to be inhibited due to the accelerating and intense colonization of the host by the pathogen (Esquerrt-tugaye et al., 1979), that illustrates the lower expression of this gene model in infected plant seedlings compared with seedlings grow in controlled conditions (Figure 7). Some LR genes were mapped on chromosome 6A such as *Lr64* (Kolmer et al., 2019), *Lr56* (Marais et al., 2006), and *Lr62* (Marais et al., 2009). Unfortunately, these genes were not included in the near isogenic lines evaluation due to seed lack. However, no previous studies postulated the presence of any of these genes in the Egyptian wheat germplasm (Draz et al., 2015, 2021; Atia et al., 2021; Draz and Abd El-Kreem, 2021). Furthermore, non of the 15 identified effective genes was mapped on this chromosome. The presence of significant markers associated with LR seedling resistance on 6A chromosome was reported previously (Juliana et al., 2018). Based on our findings and previous studies, we can conclude that 6A chromosome carries an unknown major gene/s which is effective against many LR races existing in the Egyptian fields. Therefore, in depth understanding of makers identified on this chromosome is needed to improve LR resistance in the Egyptian wheat germplasm.

The four markers on chromosomes 6D that were identified as common across the three experiments could be considered as a good source to obtain broad-spectrum resistance against most of *Pt* races in the Egyptian filed. These four markers were found to be harboring with two different gene models, TraesCS6D02G386900 (three markers in complete LD) and TraesCS6D02G389600 (one marker). The direct relationship found between TraesCS6D02G386900 gene model and disease resistance confirms our results. Furthermore, the higher expression of this gene model under disease conditions as well as its functional annotation (RPM1 disease resistance protein) confirm our findings. Previous studies identified significant MTA against MBRJ-LR race located in 462.6 Mbp on this chromosome which is very near from our identified gene models (464.1 Mbp; Fatima et al., 2020). The MBRJ was not included in our study as it was not reported to exist in the Egyptian fields. Due to the near distance between our identified gene model and the previous findings, we can conclude that 6D chromosome seems to carry important genes which are effective against a wide range of *Pt* races. The second common gene model identified on 6D chromosome (TraesCS6D02G389600) was found to control the disease resistance in wheat indirectly by controlling signal peptide peptidase-like protein, one of wheat secret proteins which enable it to resist foliar disease like LR (Zhou et al., 2020). It was reported that rust pathogen destroys the host cell by producing hydrolase enzyme that degrades the host plant proteins (Pinter et al., 2019). As peptidase-like proteins is one of the key players in plant development by regulating protein functions and breakdown the storage compounds in seeds (Santamaría et al., 2014), lower expression of their gene expression such as TraesCS6D02G389600 is expected under disease conditions (Figure 7).

Based on our findings and previous studies, we can conclude that both gene models identified on chromosome 6D, and their related markers are very important to provide broad-spectrum resistance in wheat. Looking for the results of the isogenic lines, none of the identified resistance genes was mapped on chromosome 6D. However 6D chromosomes was reported as a good source of resistance genes against many foliar disease including LR (Salina et al., 2015). Based on McIntosh et al. (1995) atlas, no *Lr* genes were mapped on 6D, except *Lr38* which provides IT = 0. Unfortunately, we could not include this gene in our evaluation due to the lack in seeds of its line. However, previous study reported the susceptibility of this gene against the Egyptian races of LR (El-Orabey et al., 2020). Therefore, we can confirm that chromosomes 6D carries unknown resistant genes which provides broad-spectrum resistance to almost all *Pt* Egyptian races. Identifying and understanding the genetic control of LR in this region will provide important sources of broad-spectrum resistance.

Remarkably, all the common SNPs associated with leaf rust in the three experiments had *R*^2^ > 10% indicating that these SNPs can be considered major QTLs controlling resistance to leaf rust. Moreover, these markers can be converted to Kompetitive allele specific PCR (KASP) marker and used for marker-assisted selection (MAS) as they were associated with resistance to different *Pt* populations. Using three different markers sets generated from different genotyping methods was very useful for detecting as many as genomic regions associated with leaf rust resistance. The markers used in this study covered a lot of genomic regions in the wheat three genomes.

### Selection of Superior Genotypes With Broad-Spectrum Resistance Against Egyptian Leaf Rust Races

It was reported that LR resistance began to be broken down in the Egyptian wheat germplasm. In our recent study, 35 Egyptian wheat cultivars and breeding lines were evaluated including some resistant genotypes such as Misr2, Sids-13, Sids-14, Sakha-94, Sohag-5 (Atia et al., 2021; Draz et al., 2021). Based on our evaluation all the resistant genotypes provided moderate to susceptible reaction against the studied LR races which confirms the losing of resistance in Egyptian wheat and the urgent needs for further sources of resistance. The selected genotypes presented in Table 2 are a good source to improve LR resistance in the Egyptian wheat genetic germplasm. This could be concluded by the identified immune genotypes (PI_381963 and PI_595213) which had higher resistance levels than the selected Egyptian genotypes. Furthermore, the Iranian genotype (PI_381963) was found to carry almost all the targeted alleles of the significant markers identified in the current studies (Figure 8). This number was greater than the number of targeted alleles represented in all the selected Egyptian genotypes. In addition, based on population structure analysis, the immune Iranian genotype (PI_381963) was in subpopulation-1 far from the resistant genotypes that located in subpopulation-2 and 3. Previous studies concluded that the high genetically distant genotypes are the best parents to be crossed in order to improve specific traits (Bertan et al., 2007; Mourad et al., 2020). Based on the high genetic distance between this Iranian genotype and the resistant Egyptian genotypes, we can suggest the Iranian genotype as a good parent to improve LR resistance in the Egyptian wheat germplasm. Unfortunately, the American immune genotype (Hutch) was not included in the GBS-SNP data set, therefore STRUCTURE analysis was not available for it. However, based on the PCA results of 9K-SNPs and DArT marker, Hutch genotype was located either in subpopulation-1 or subpopulation-2, far from two Egyptian genotypes Qadry_006 and Sohag-5. Therefore, Hutch is also considered as a good parent to improve LR resistance under the Egyptian conditions. Using the selected genotypes identified in the current study in future breeding programs will accelerate wheat germplasm not only against LR but also against other types of rust diseases existing in the Egyptian fields. Combining the analyses from phenotypic selection along with extensive genetics analysis such population structure, genetic diversity, and GWAS results promise with identify the true and most promising resistance genotypes for future breeding program. Phenotypic selection can be misled due to human errors and the large effects of the environments, however, inducing the advances in QTL detection (e.g., GWAS) and genetic diversity can have a great impact in accelerating breeding program associated with target traits (Sallam et al., 2019). This approach was applied to identify the candidate genotypes as parents for many target traits such as high grain yield (Eltaher et al., 2021a) and high resistance to disease (Mourad et al., 2018b; Abou-Zeid and Mourad, 2021; Bhavani et al., 2021; Eltaher et al., 2021b).

## Conclusion

In conclusion, the high variation in the tested 198-spring wheat diverse genotypes against different Egyptian *Pt* races suggesting the possibility to select broad-spectrum resistant genotypes against leaf rust using the current plant materials. Furthermore, the different genotyping marker sets used in this study are covering different parts from the wheat genome hence enable the genome-wide screening that identifies different genomic regions associated with LR resistance. The identified 48 gene models harboring the significant markers represent the possibility of obtaining broad-spectrum resistance by pyramiding all the significant markers in one genotype. However, the common four gene models among the three experiments and the Sakha’s experiments reducing efforts needed to obtain broad-spectrum resistance. The selected genotypes identified in the current study provides good sources of resistance to the different *Pt* races in the Egyptian wheat germplasm due to their highly resistant and high genetic distance among them.

## Data Availability Statement

The original contributions presented in the study are included in the article/Supplementary Materials, further inquiries can be directed to the corresponding author.

## Author Contributions

AM designed the experiment, performed the genetic and phenotyping analysis, discussed the results, and drafted the manuscript. SE, ID, and GO performed the phenotyping for leaf rust resistance. AB reviewed the manuscript. The authors agreed to be accountable for the content of the work. All authors contributed to the article and approved the submitted version.

## Funding

The publication of this article was funded by the Deutsche Forschungsgemeinschaft (DFG, German Research Foundation) – 801 HE 9114/1-1.

## Conflict of Interest

The authors declare that the research was conducted in the absence of any commercial or financial relationships that could be construed as a potential conflict of interest.

## Publisher’s Note

All claims expressed in this article are solely those of the authors and do not necessarily represent those of their affiliated organizations, or those of the publisher, the editors and the reviewers. Any product that may be evaluated in this article, or claim that may be made by its manufacturer, is not guaranteed or endorsed by the publisher.

## Footnotes

^1^ https://npgsweb.ars-grin.gov/gringlobal/search.aspx

^2^ https://wheat.pw.usda.gov/cgi-bin/GG3/browse.cgi

^3^ https://knetminer.rothamsted.ac.uk/Triticum_aestivum/

^4^ http://www.wheat-expression.com/
