# Supplementary material for: Genome-Wide Screening of Broad-Spectrum Resistance to Leaf Rust (Puccinia triticina Eriks) in Spring Wheat (Triticum aestivum L.)
Source: Front Plant Sci. 2022 Jun 22;13:921230. doi: 10.3389/fpls.2022.921230 (PMC9258335; doi:10.3389/fpls.2022.921230)
Supplement: Supplementary file 1 [file Presentation_1.PPTX]

## Slide 1
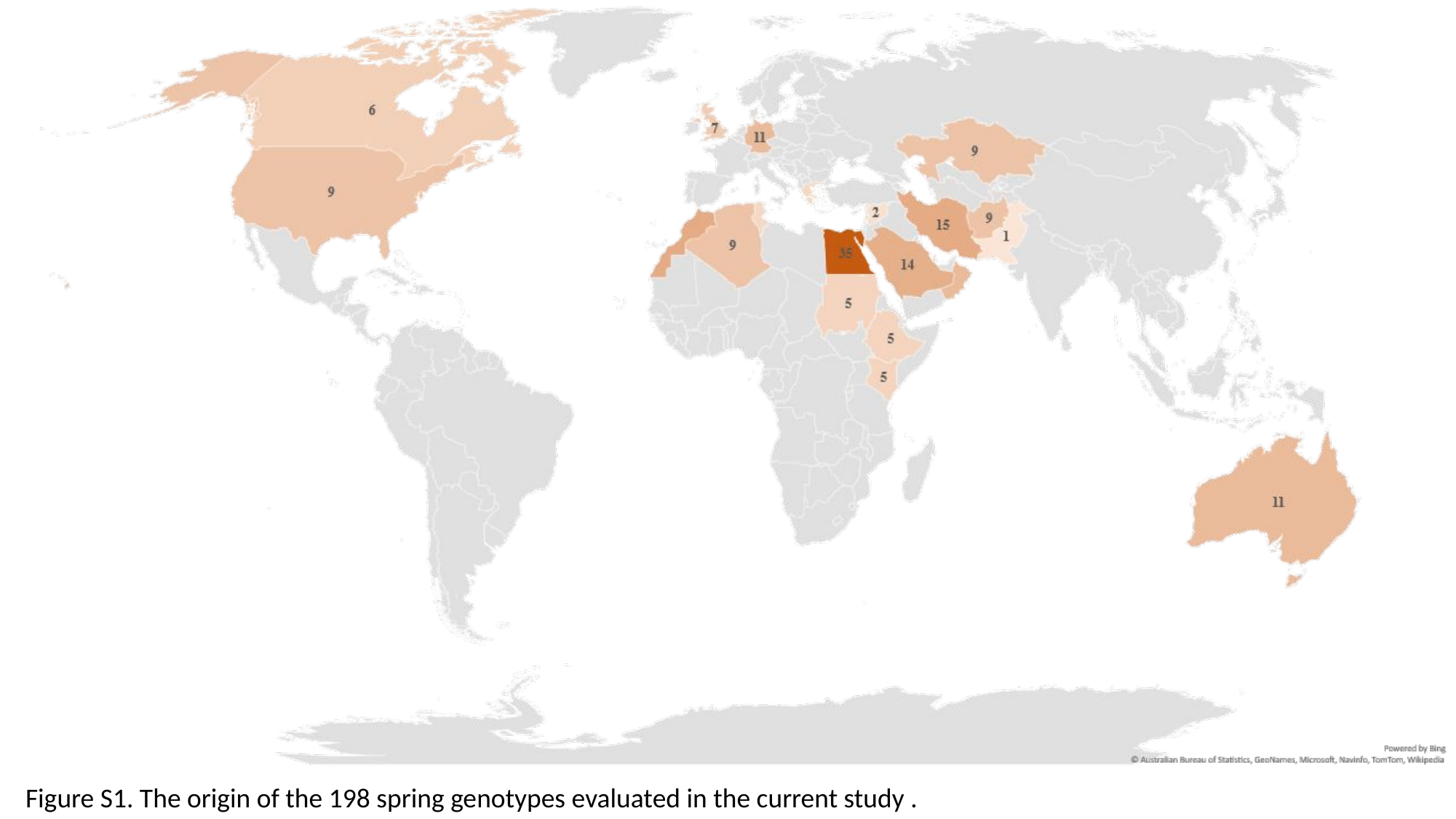

Figure S1. The origin of the 198 spring genotypes evaluated in the current study .

## Slide 2
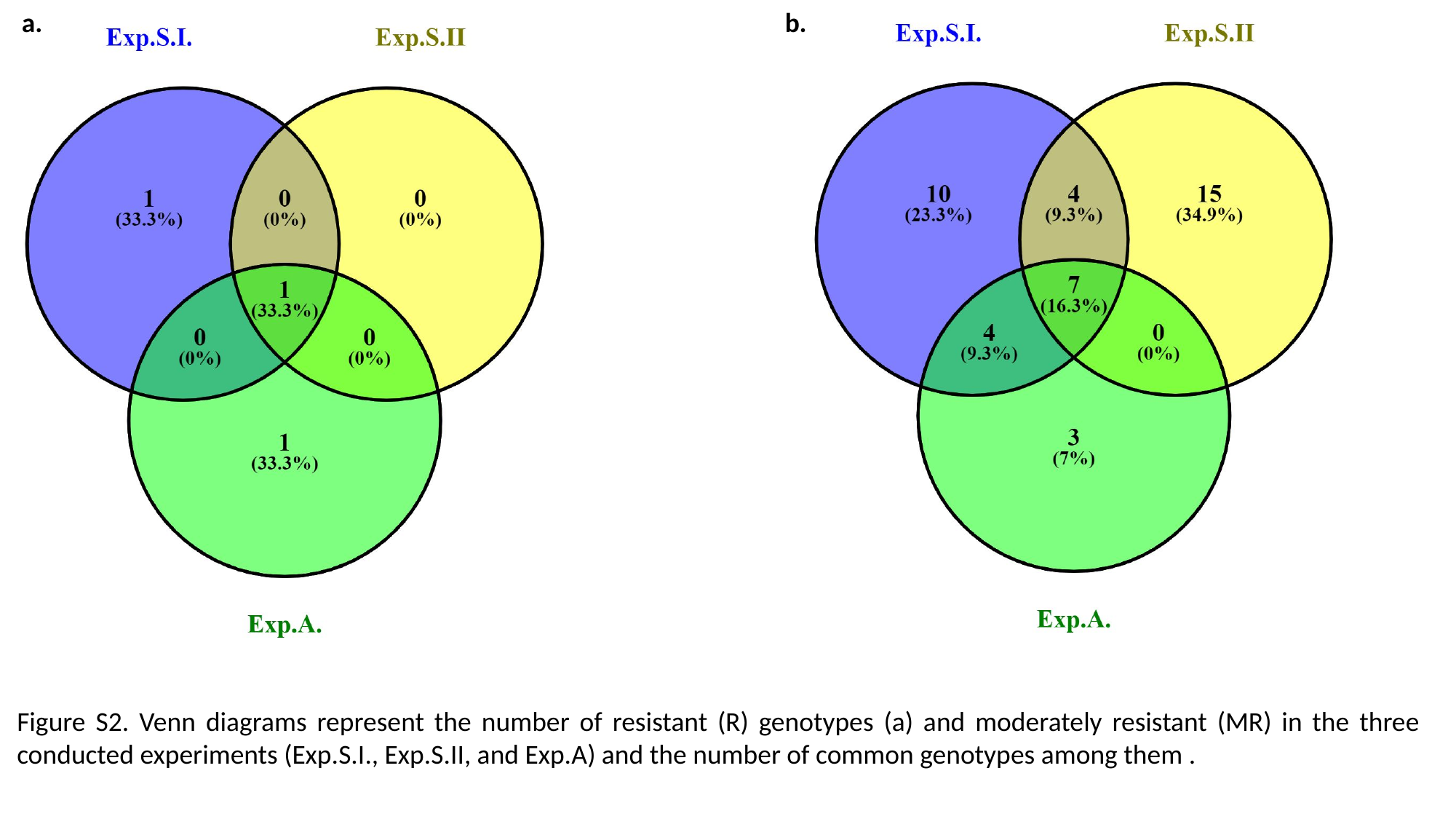

a.
b.
Figure S2. Venn diagrams represent the number of resistant (R) genotypes (a) and moderately resistant (MR) in the three conducted experiments (Exp.S.I., Exp.S.II, and Exp.A) and the number of common genotypes among them .

## Slide 3
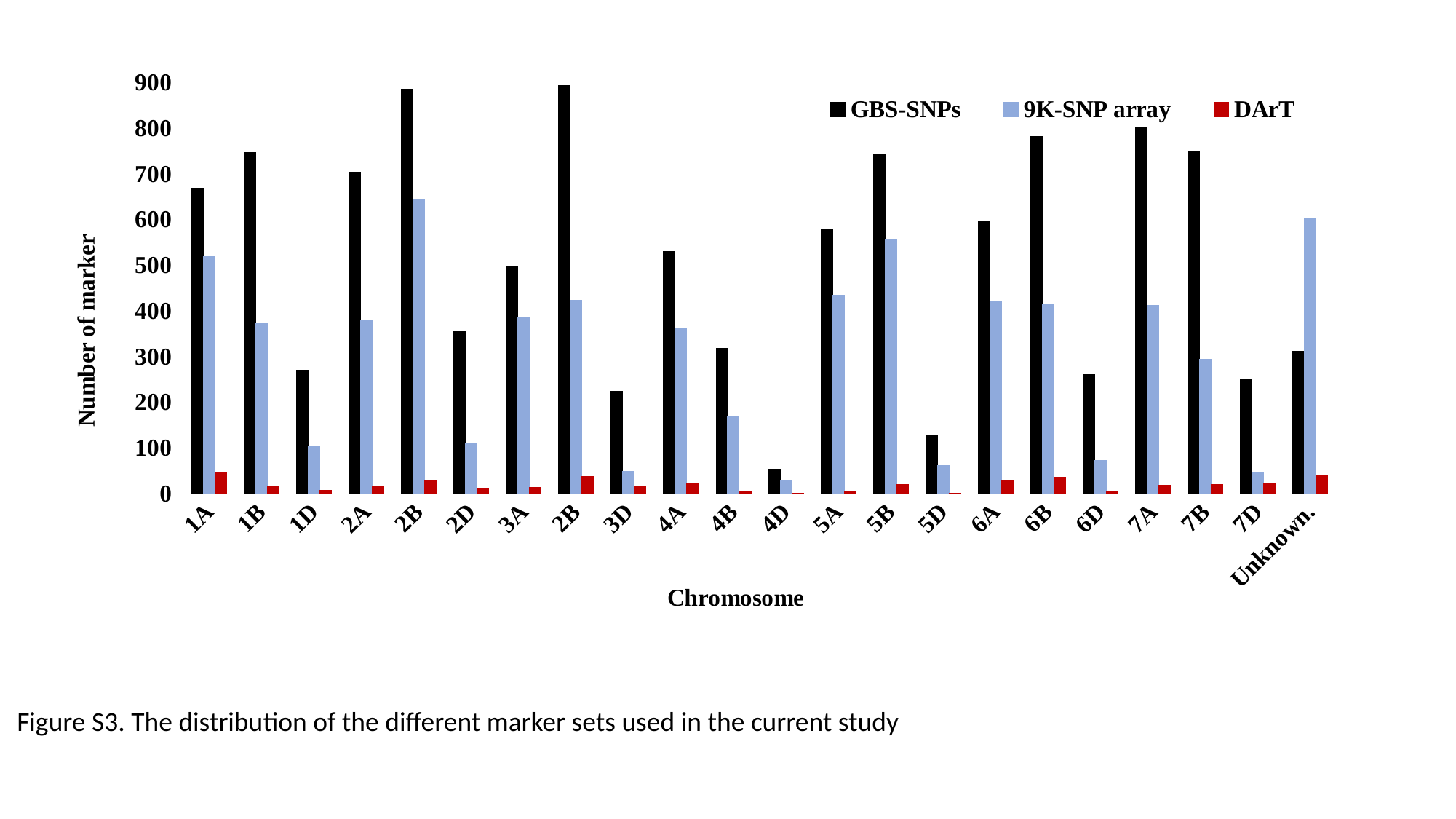

### Chart
| Category | GBS-SNPs | 9K-SNP array | DArT |
|---|---|---|---|
| 1A | 669.0 | 521.0 | 46.0 |
| 1B | 748.0 | 375.0 | 16.0 |
| 1D | 271.0 | 105.0 | 8.0 |
| 2A | 705.0 | 380.0 | 17.0 |
| 2B | 886.0 | 645.0 | 29.0 |
| 2D | 356.0 | 112.0 | 12.0 |
| 3A | 498.0 | 386.0 | 15.0 |
| 2B | 894.0 | 424.0 | 38.0 |
| 3D | 224.0 | 50.0 | 17.0 |
| 4A | 531.0 | 361.0 | 23.0 |
| 4B | 318.0 | 170.0 | 6.0 |
| 4D | 54.0 | 29.0 | 1.0 |
| 5A | 580.0 | 435.0 | 5.0 |
| 5B | 742.0 | 557.0 | 21.0 |
| 5D | 128.0 | 63.0 | 2.0 |
| 6A | 597.0 | 423.0 | 31.0 |
| 6B | 783.0 | 414.0 | 37.0 |
| 6D | 262.0 | 74.0 | 7.0 |
| 7A | 803.0 | 413.0 | 19.0 |
| 7B | 751.0 | 295.0 | 21.0 |
| 7D | 252.0 | 47.0 | 24.0 |
| Unknown. | 313.0 | 604.0 | 42.0 |Figure S3. The distribution of the different marker sets used in the current study

## Slide 4
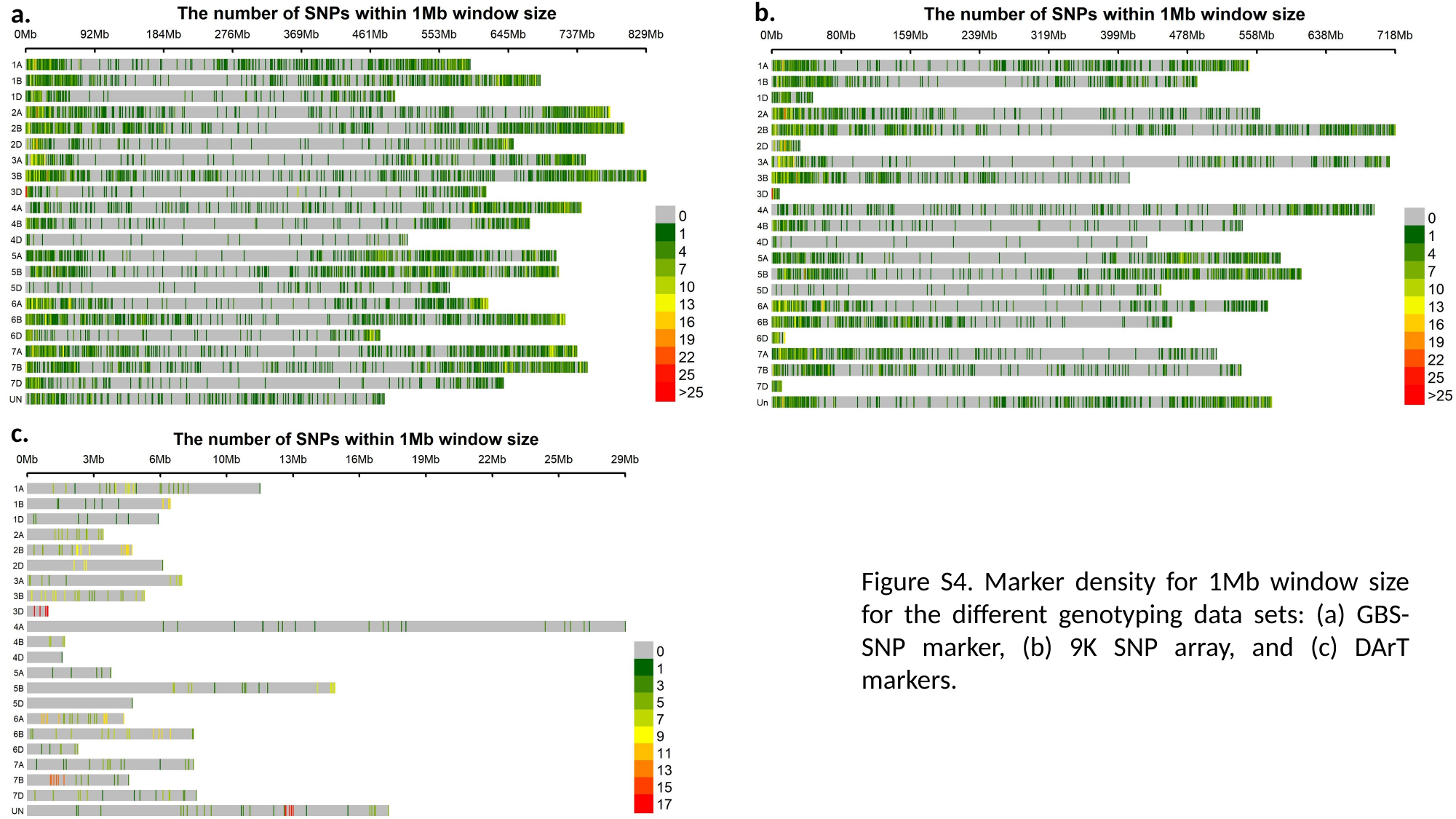

b.
a.
c.
Figure S4. Marker density for 1Mb window size for the different genotyping data sets: (a) GBS-SNP marker, (b) 9K SNP array, and (c) DArT markers.

## Slide 5
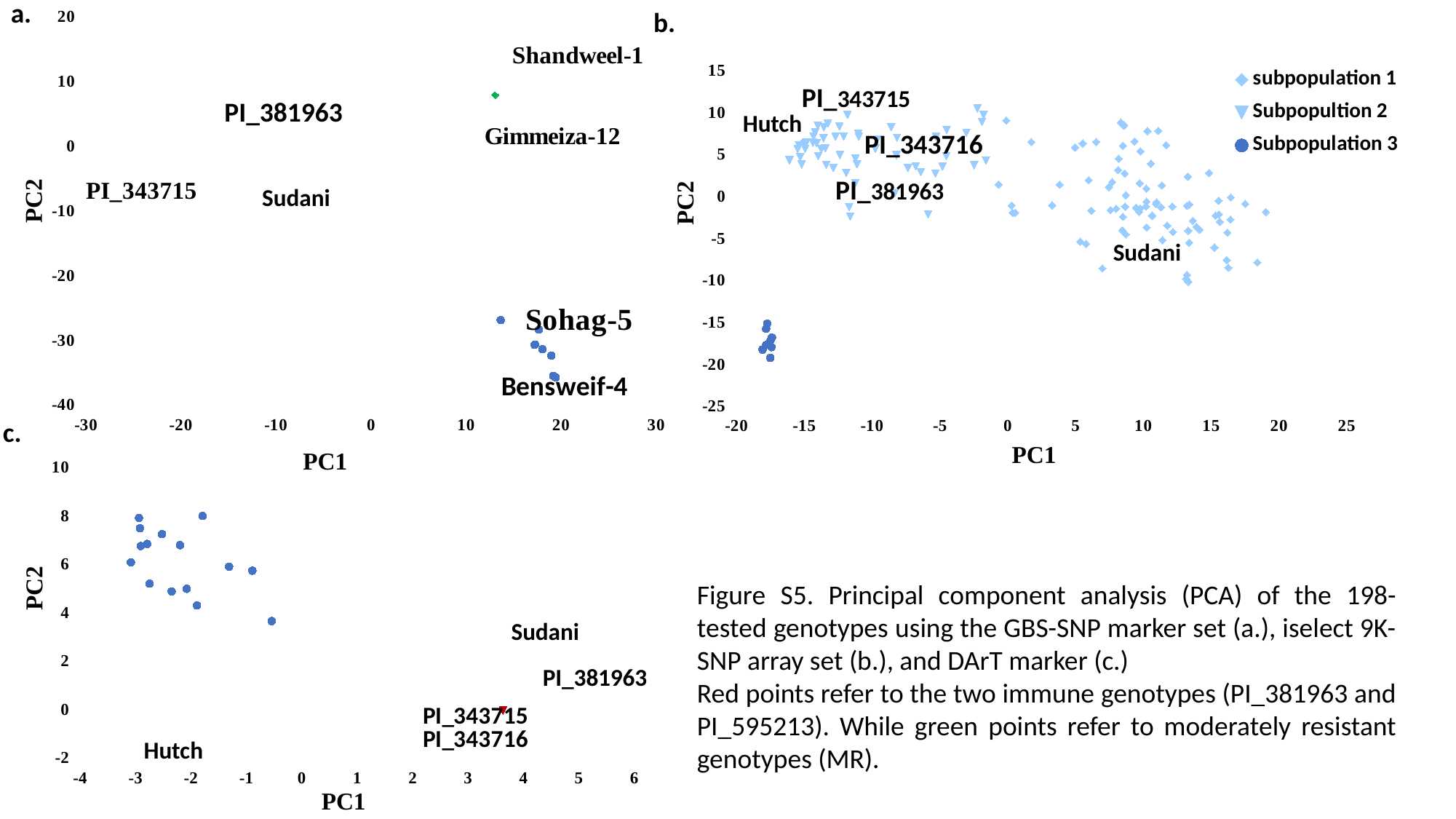

### Chart
| Category | | | |
|---|---|---|---|b.
a.
### Chart
| Category | | | |
|---|---|---|---|PI_343715
PI_381963
Hutch
PI_343716
PC2
PC2
PI_381963
Sudani
Sudani
c.
### Chart
| Category | | | |
|---|---|---|---|PC1
PC1
PC2
Figure S5. Principal component analysis (PCA) of the 198-tested genotypes using the GBS-SNP marker set (a.), iselect 9K-SNP array set (b.), and DArT marker (c.)
Red points refer to the two immune genotypes (PI_381963 and PI_595213). While green points refer to moderately resistant genotypes (MR).
Sudani
PI_381963
Hutch
PC1

## Slide 6
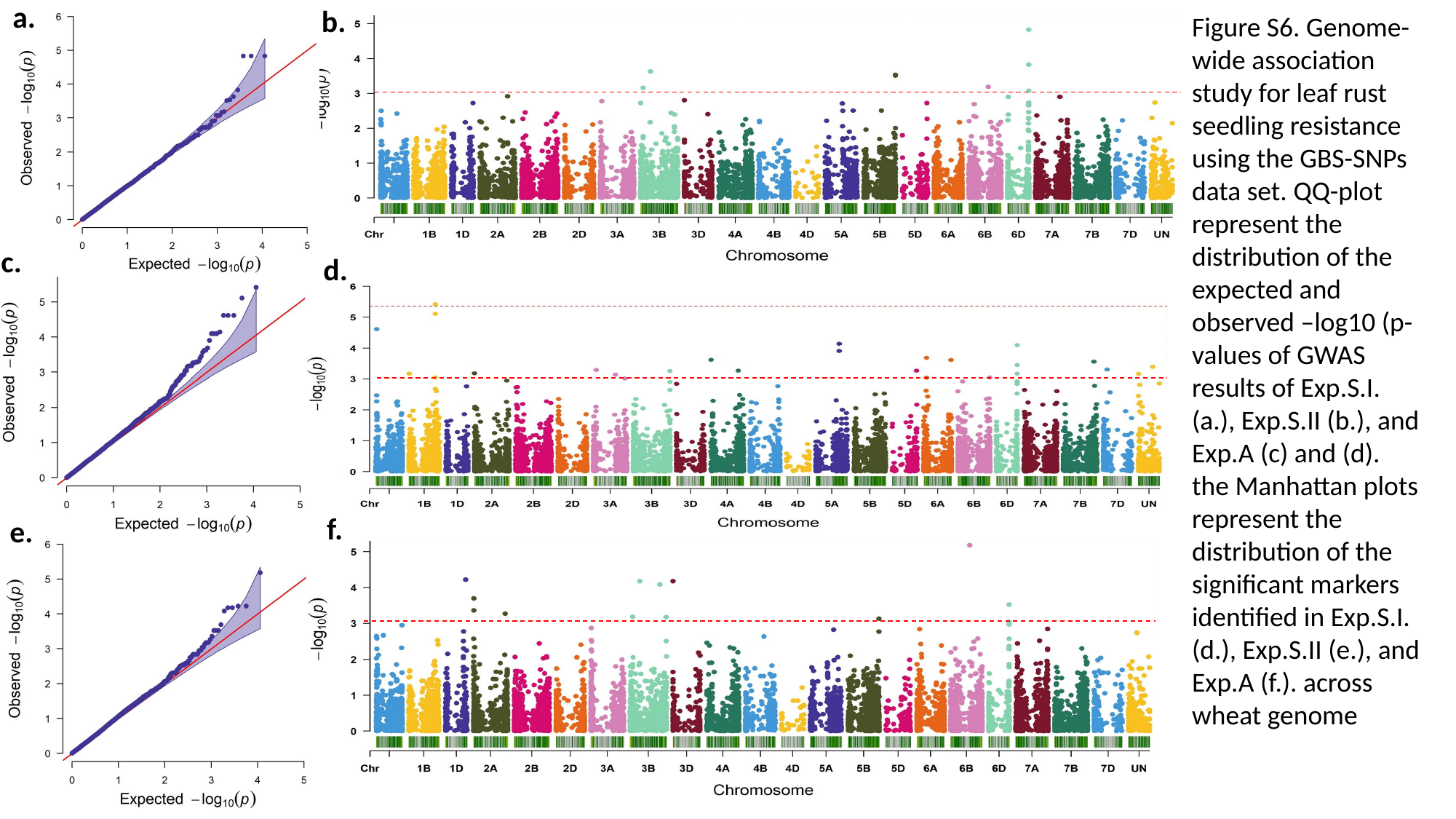

a.
b.
Figure S6. Genome-wide association study for leaf rust seedling resistance using the GBS-SNPs data set. QQ-plot represent the distribution of the expected and observed –log10 (p-values of GWAS results of Exp.S.I. (a.), Exp.S.II (b.), and Exp.A (c) and (d). the Manhattan plots represent the distribution of the significant markers identified in Exp.S.I. (d.), Exp.S.II (e.), and Exp.A (f.). across wheat genome
c.
d.
f.
e.

## Slide 7
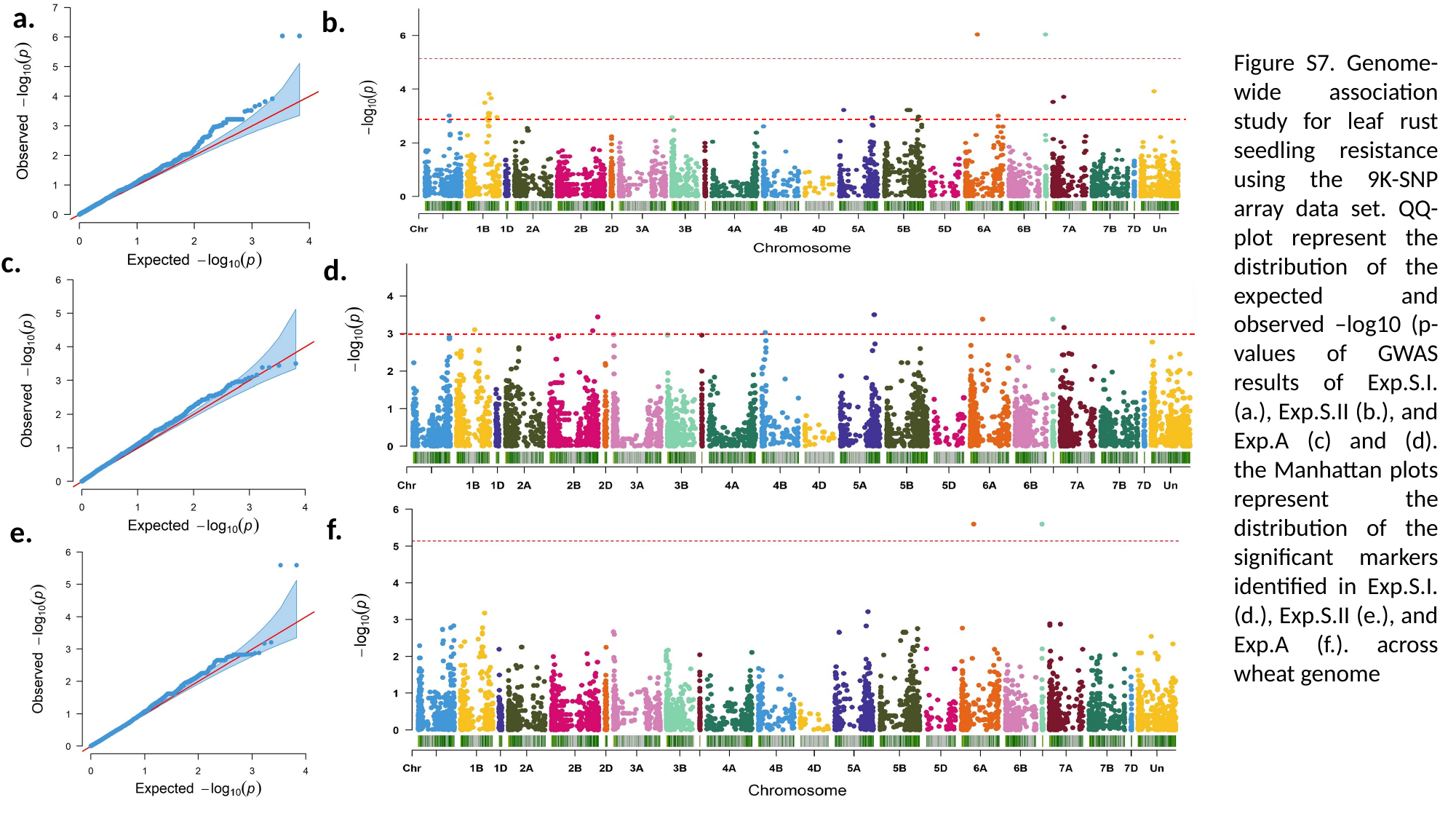

a.
b.
Figure S7. Genome-wide association study for leaf rust seedling resistance using the 9K-SNP array data set. QQ-plot represent the distribution of the expected and observed –log10 (p-values of GWAS results of Exp.S.I. (a.), Exp.S.II (b.), and Exp.A (c) and (d). the Manhattan plots represent the distribution of the significant markers identified in Exp.S.I. (d.), Exp.S.II (e.), and Exp.A (f.). across wheat genome
c.
d.
f.
e.

## Slide 8
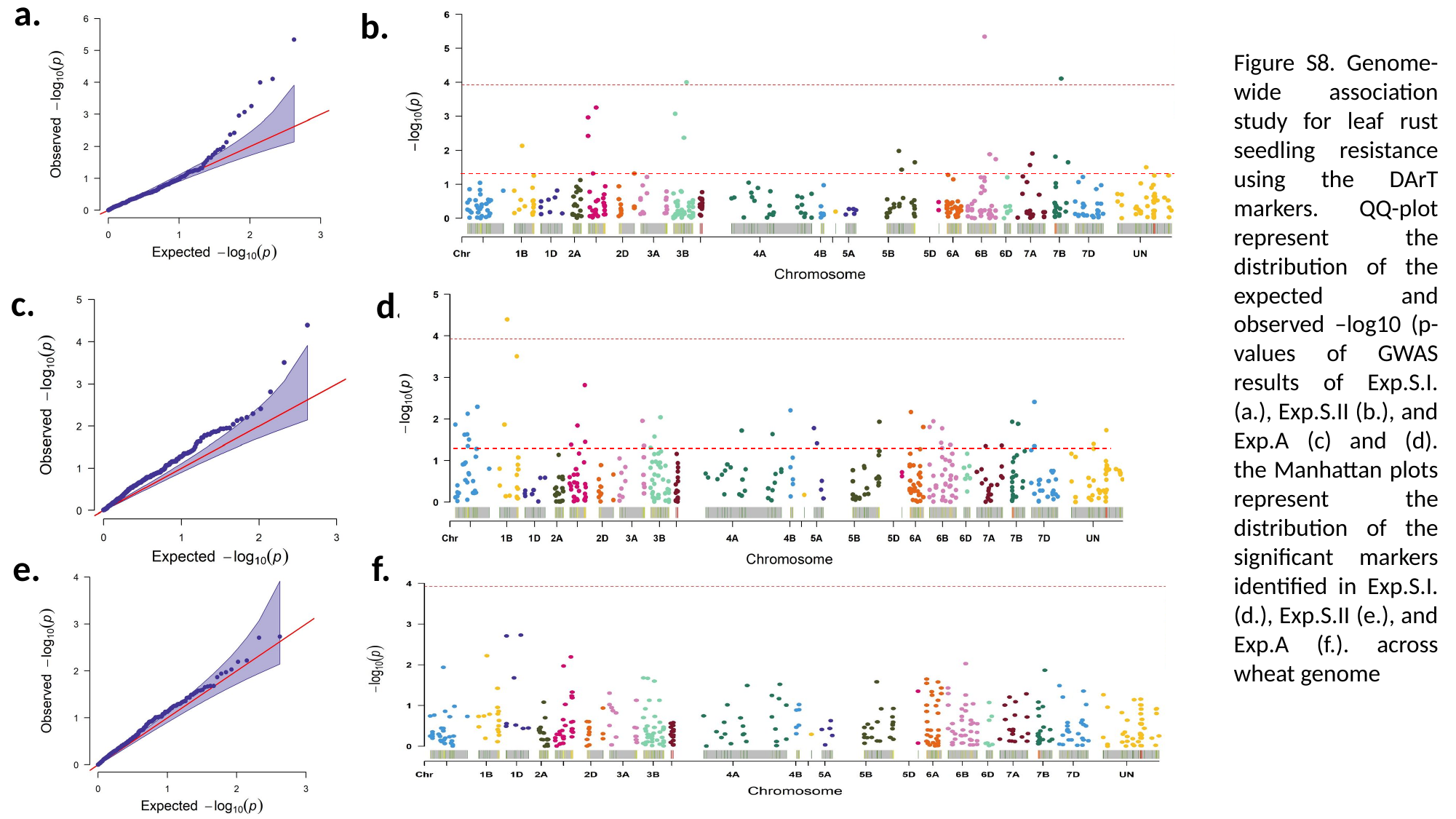

a.
b.
Figure S8. Genome-wide association study for leaf rust seedling resistance using the DArT markers. QQ-plot represent the distribution of the expected and observed –log10 (p-values of GWAS results of Exp.S.I. (a.), Exp.S.II (b.), and Exp.A (c) and (d). the Manhattan plots represent the distribution of the significant markers identified in Exp.S.I. (d.), Exp.S.II (e.), and Exp.A (f.). across wheat genome
c.
d.
e.
f.

## Slide 9
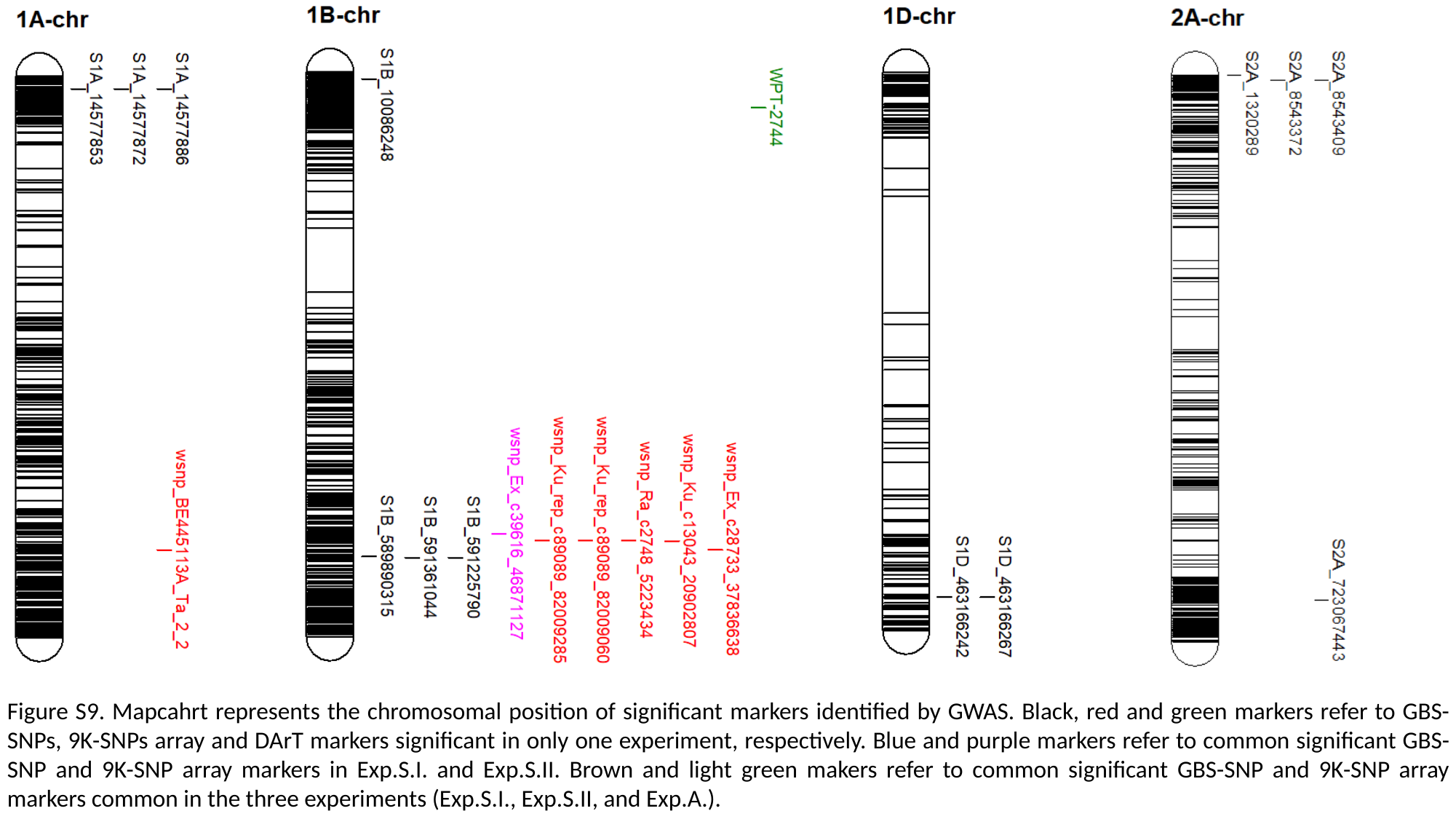

Figure S9. Mapcahrt represents the chromosomal position of significant markers identified by GWAS. Black, red and green markers refer to GBS-SNPs, 9K-SNPs array and DArT markers significant in only one experiment, respectively. Blue and purple markers refer to common significant GBS-SNP and 9K-SNP array markers in Exp.S.I. and Exp.S.II. Brown and light green makers refer to common significant GBS-SNP and 9K-SNP array markers common in the three experiments (Exp.S.I., Exp.S.II, and Exp.A.).

## Slide 10
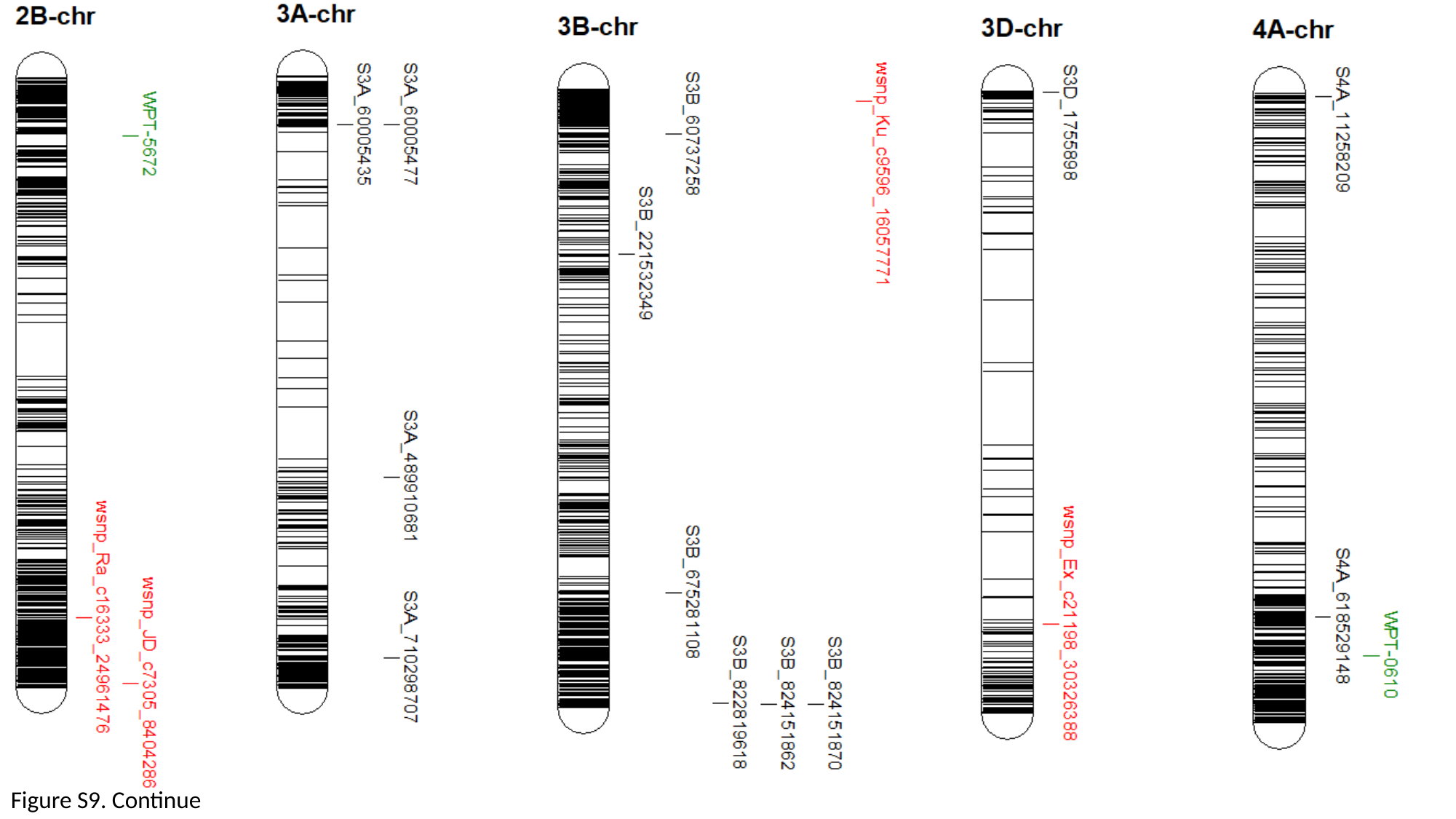

Figure S9. Continue

## Slide 11
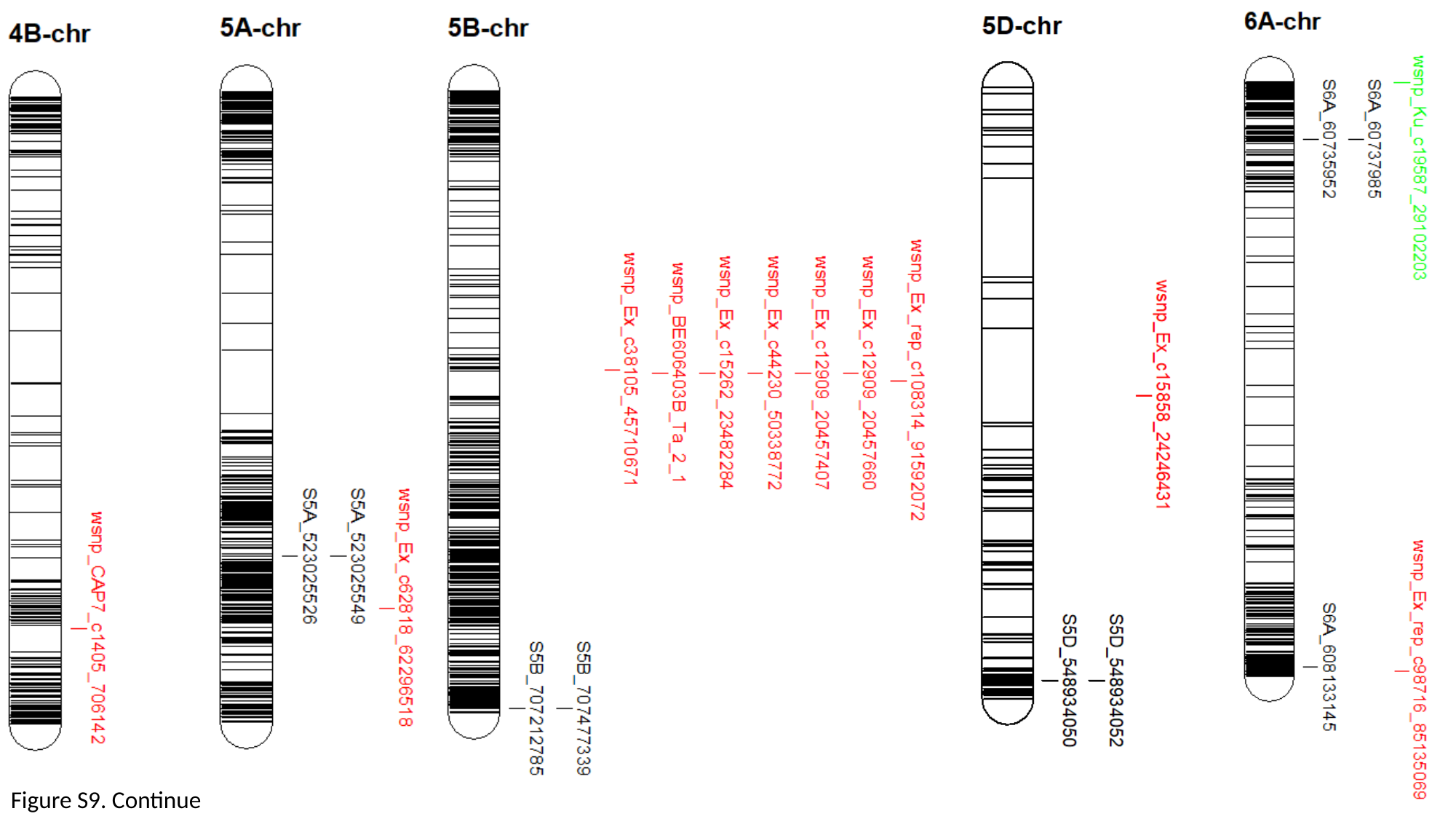

Figure S9. Continue

## Slide 12
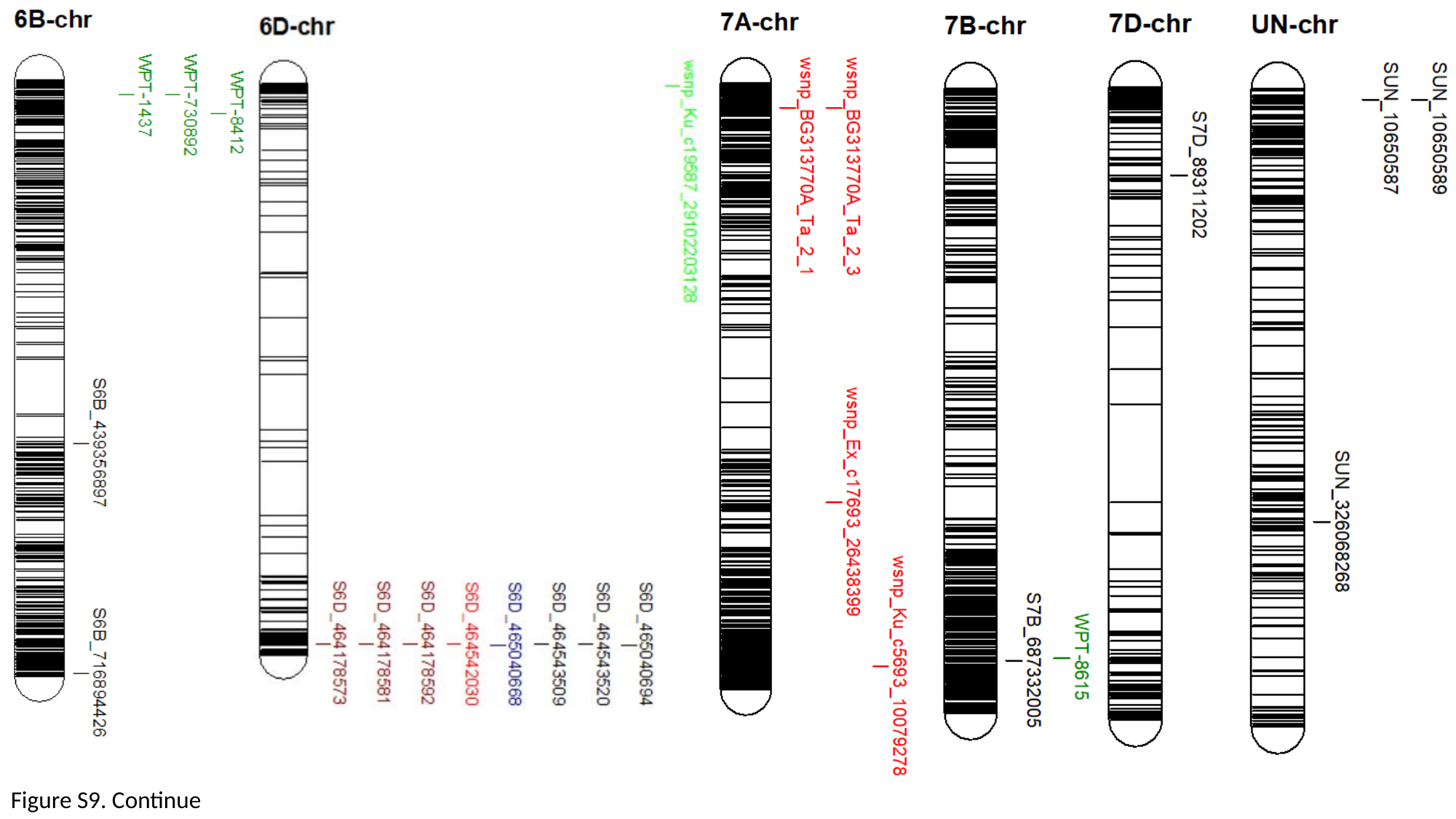

Figure S9. Continue

## Slide 13
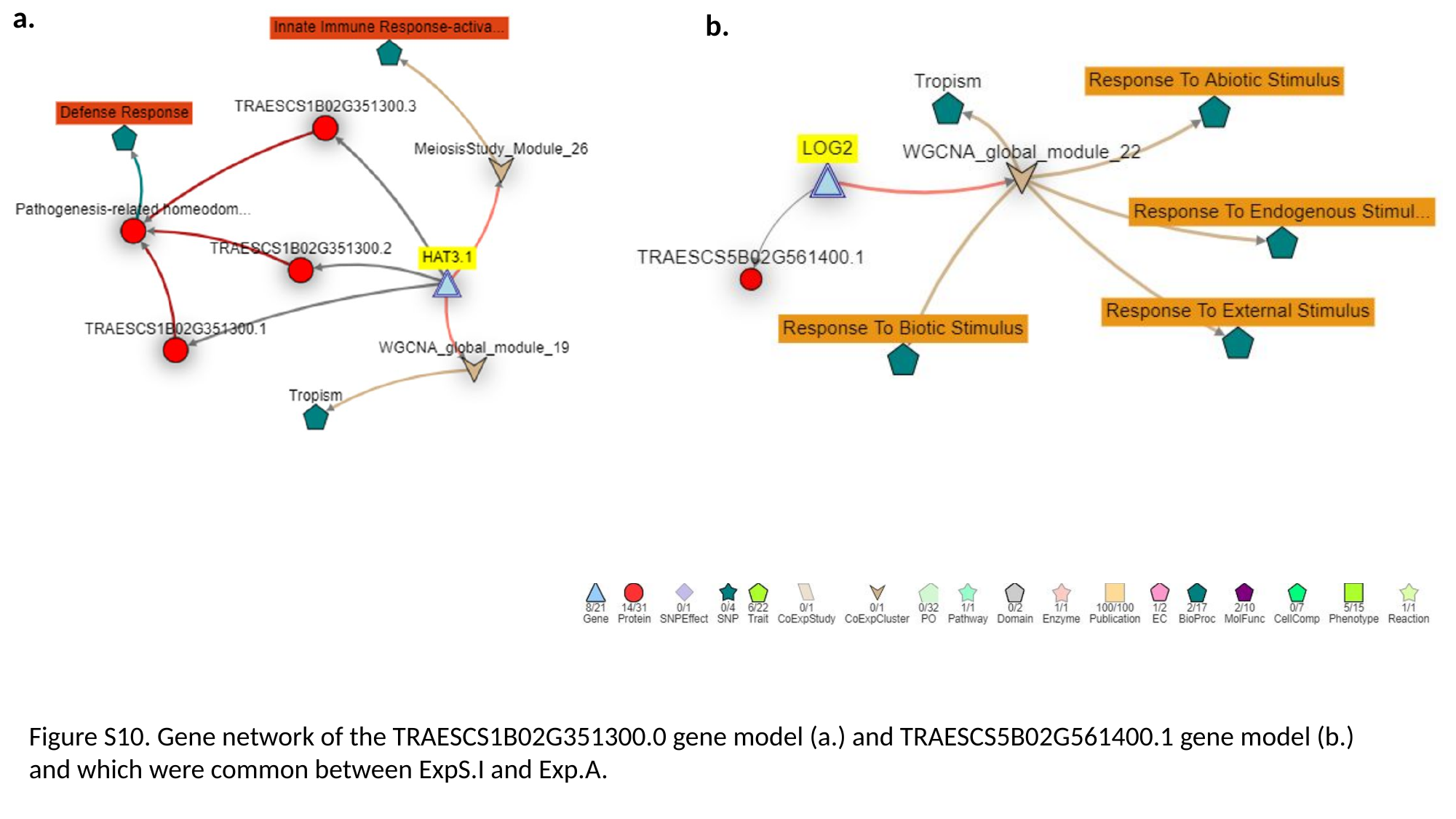

b.
a.
Figure S10. Gene network of the TRAESCS1B02G351300.0 gene model (a.) and TRAESCS5B02G561400.1 gene model (b.) and which were common between ExpS.I and Exp.A.

## Slide 14
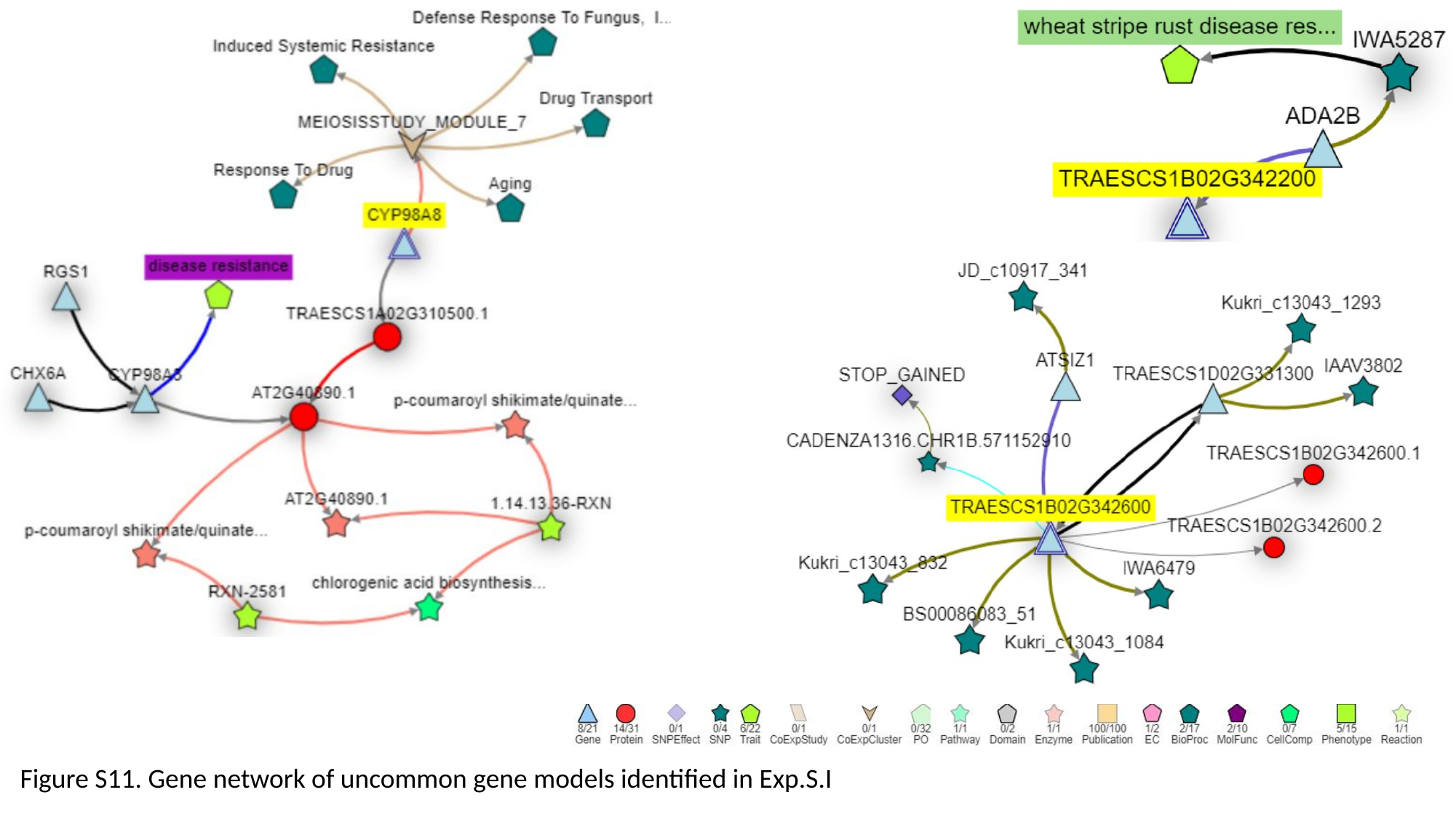

Figure S11. Gene network of uncommon gene models identified in Exp.S.I

## Slide 15
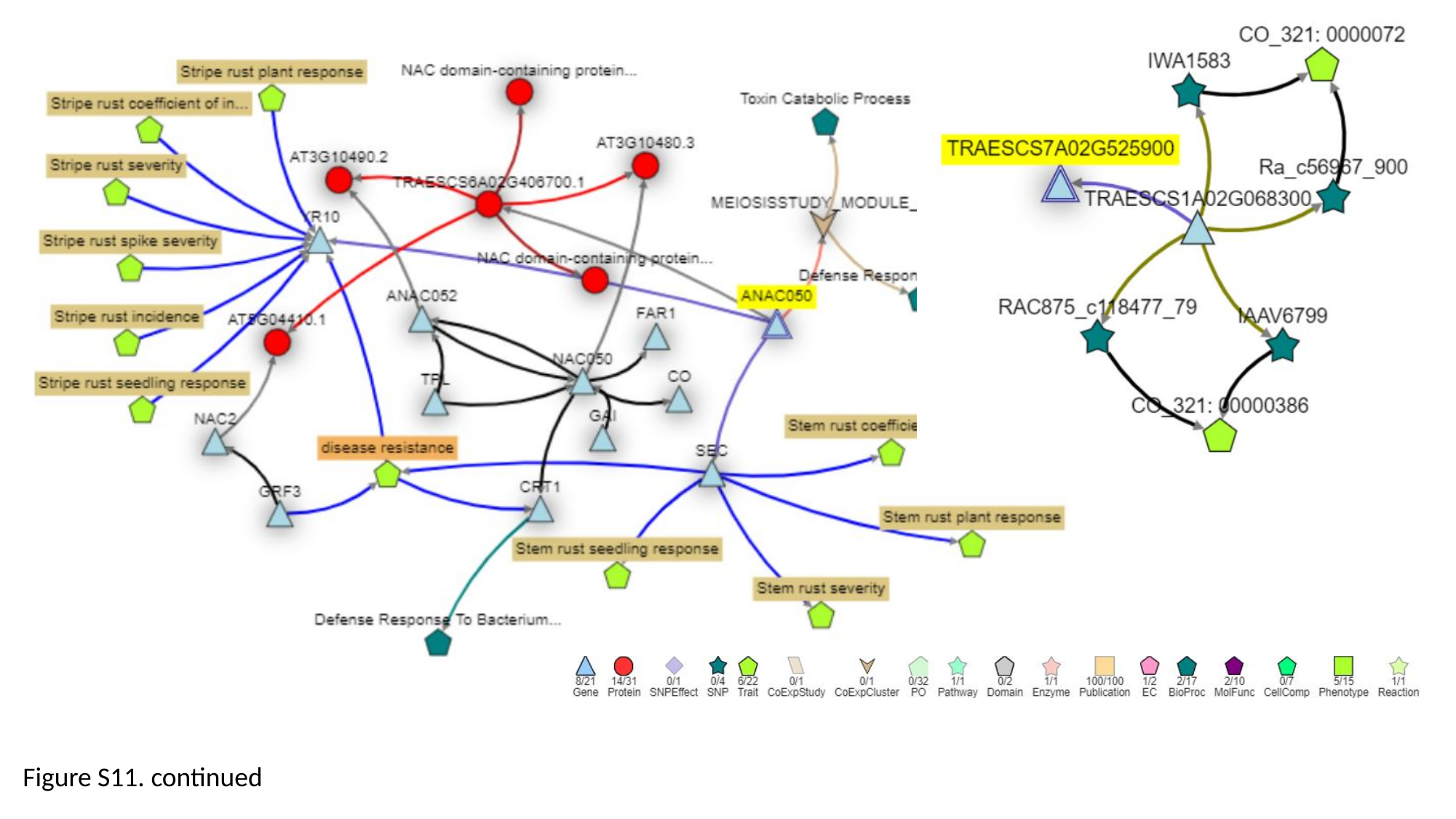

Figure S11. continued

## Slide 16
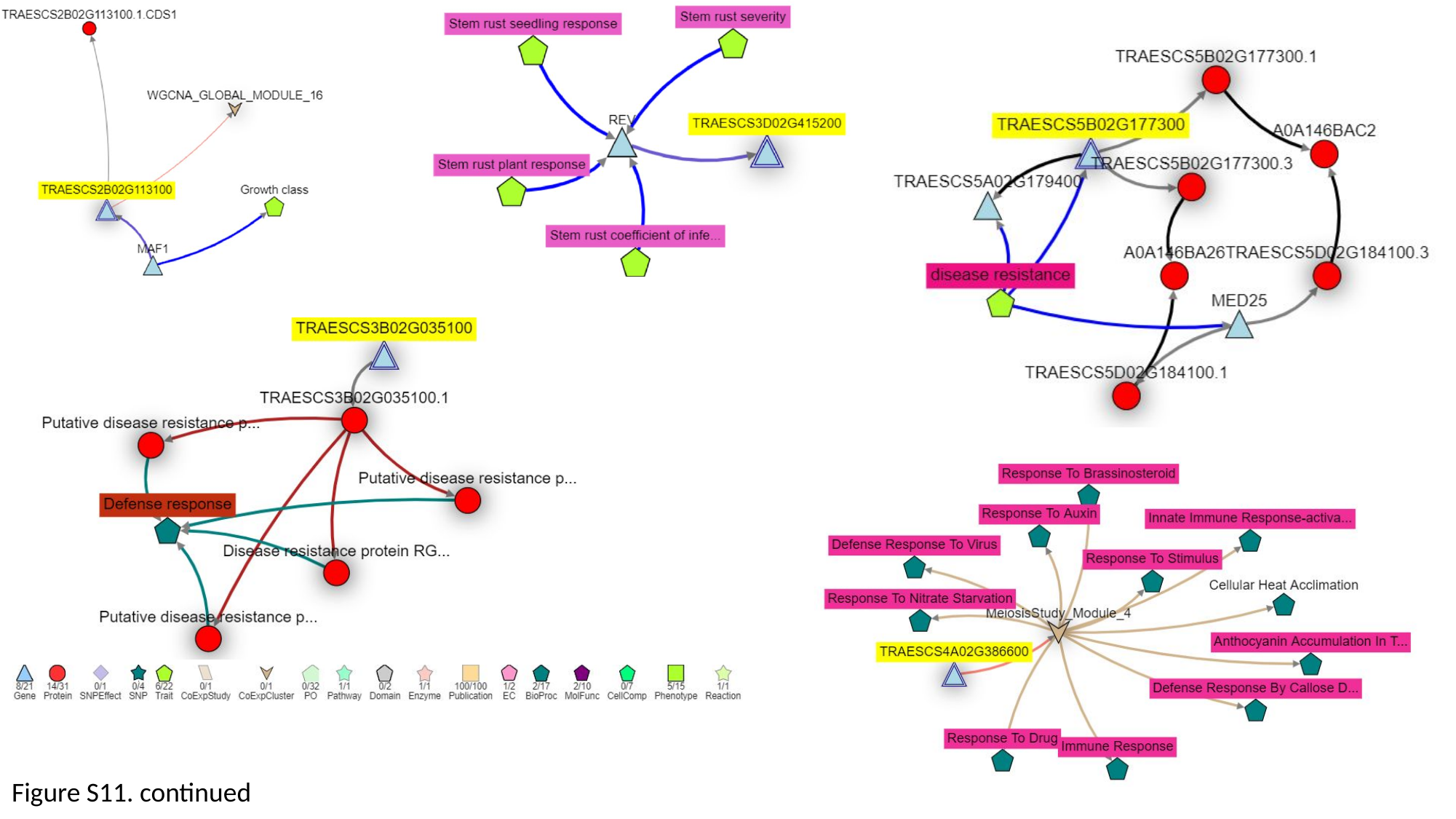

Figure S11. continued

## Slide 17
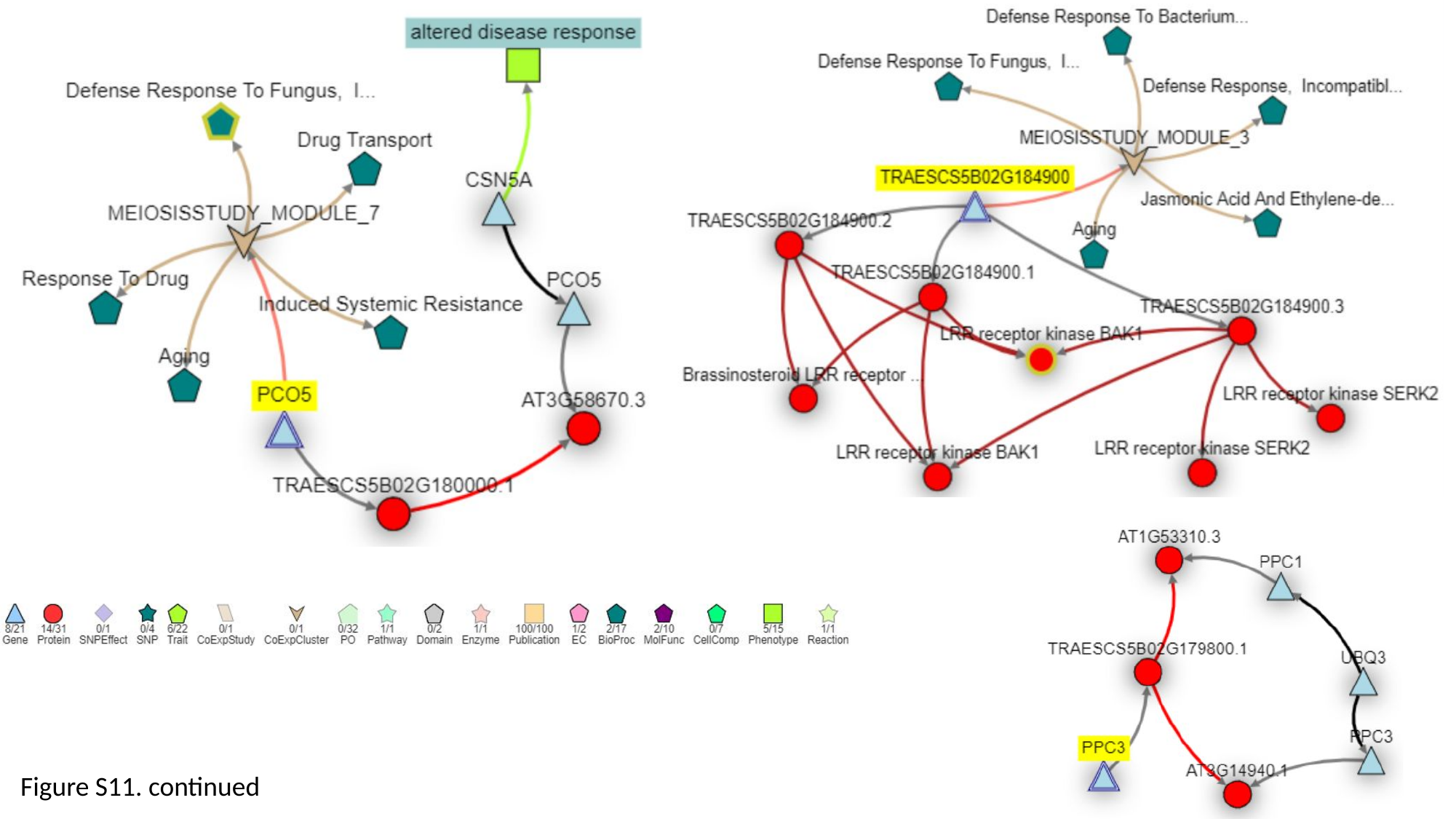

Figure S11. continued

## Slide 18
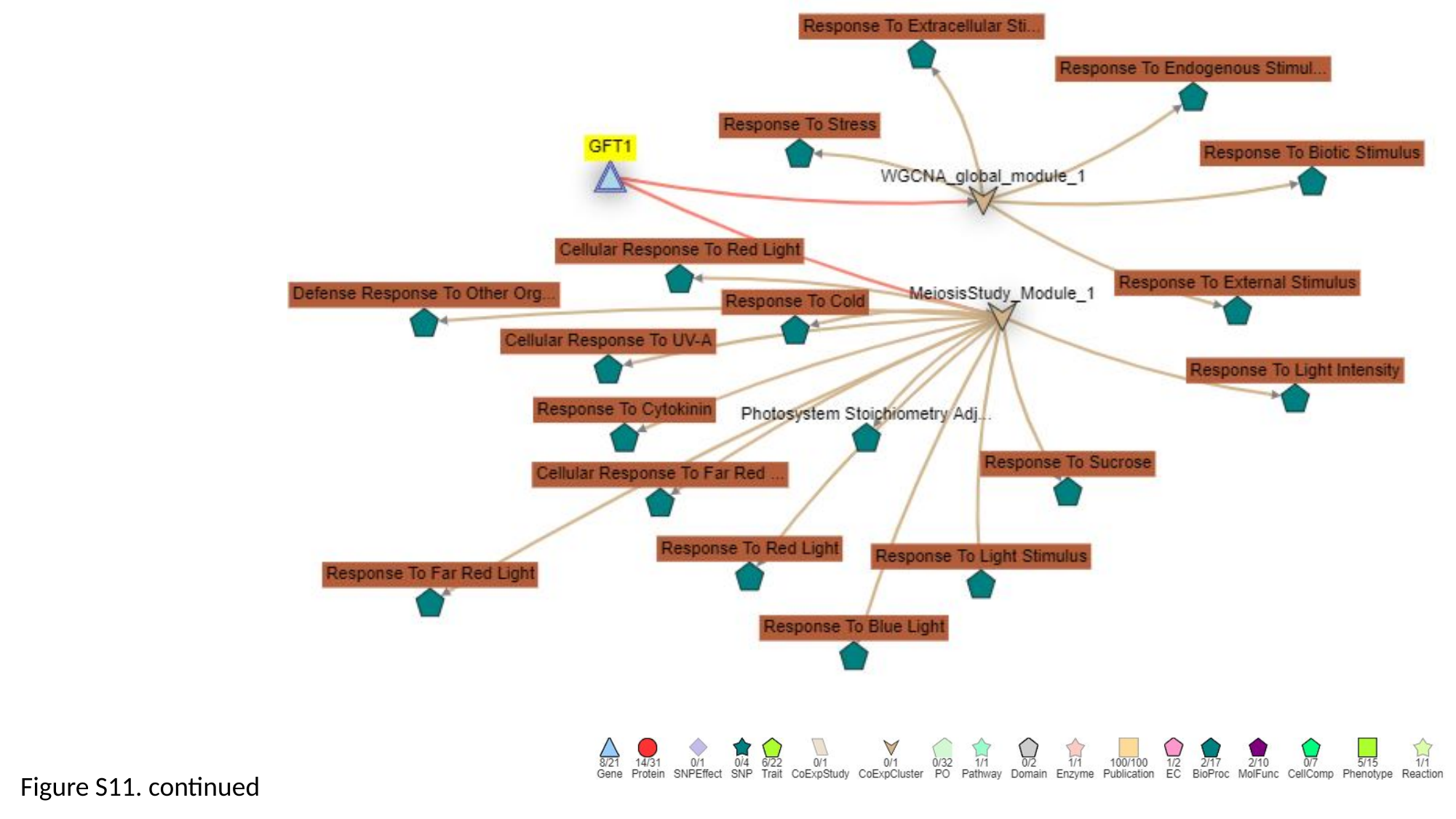

Figure S11. continued

## Slide 19
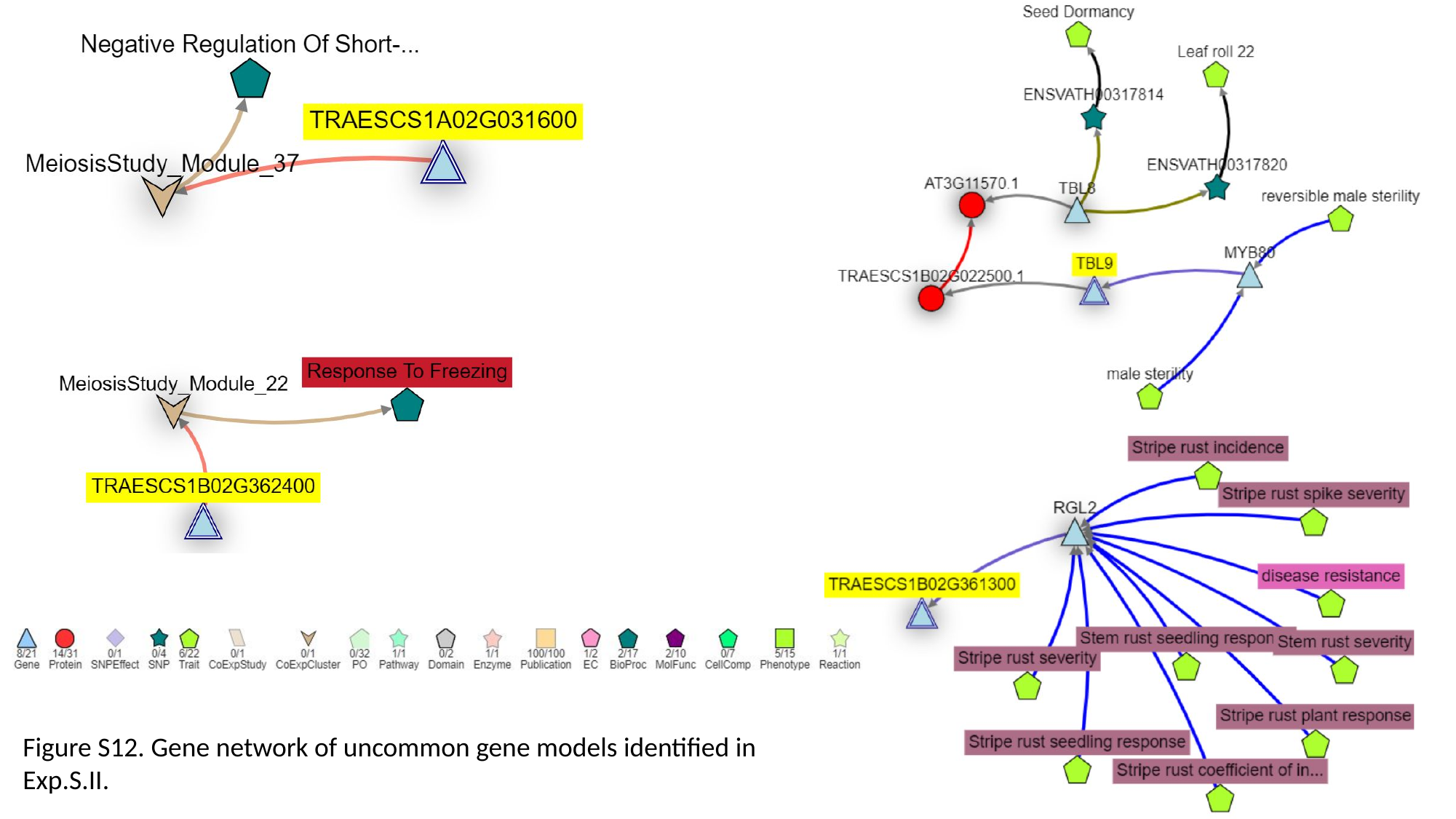

Figure S12. Gene network of uncommon gene models identified in Exp.S.II.

## Slide 20
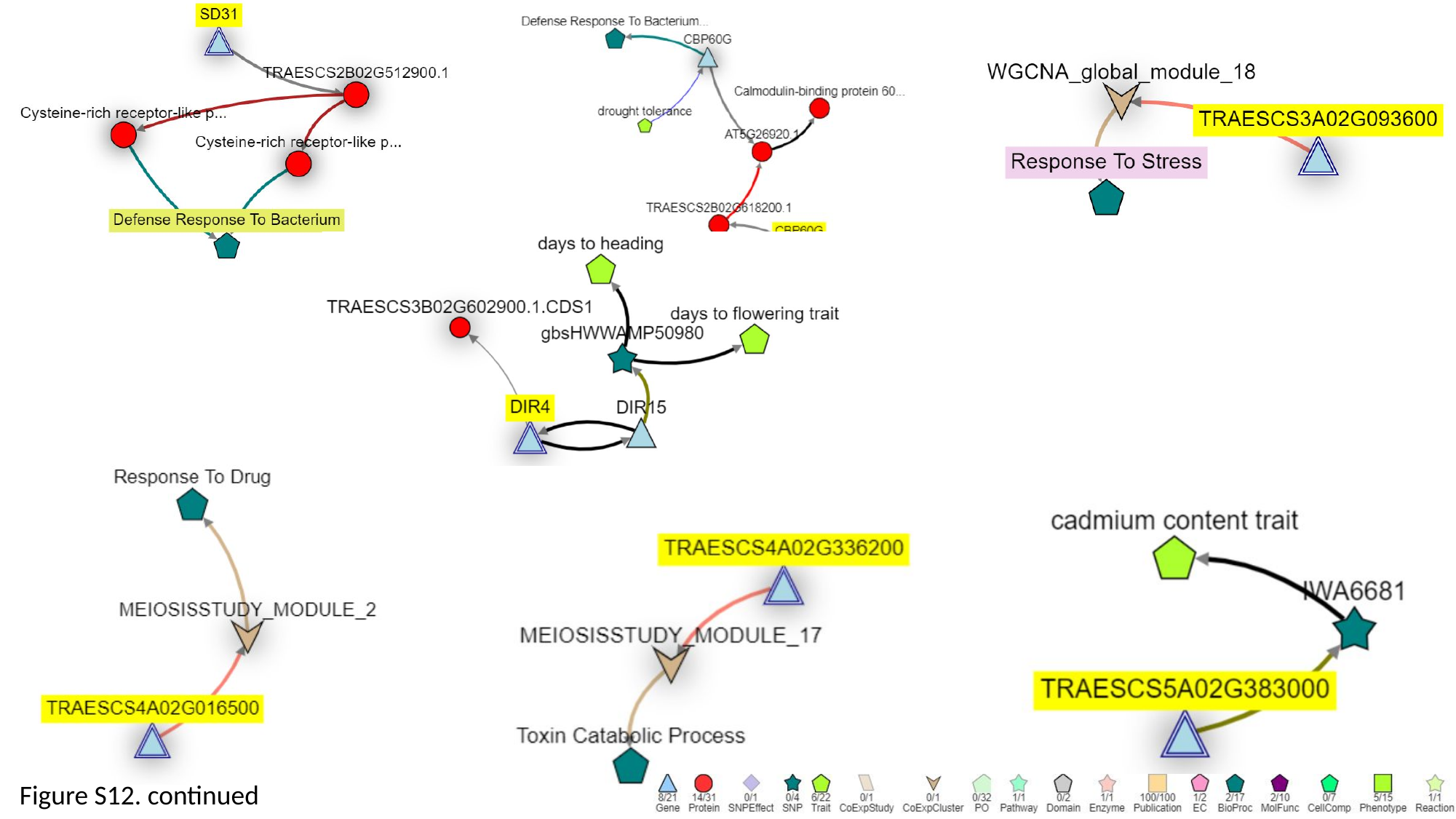

Figure S12. continued

## Slide 21
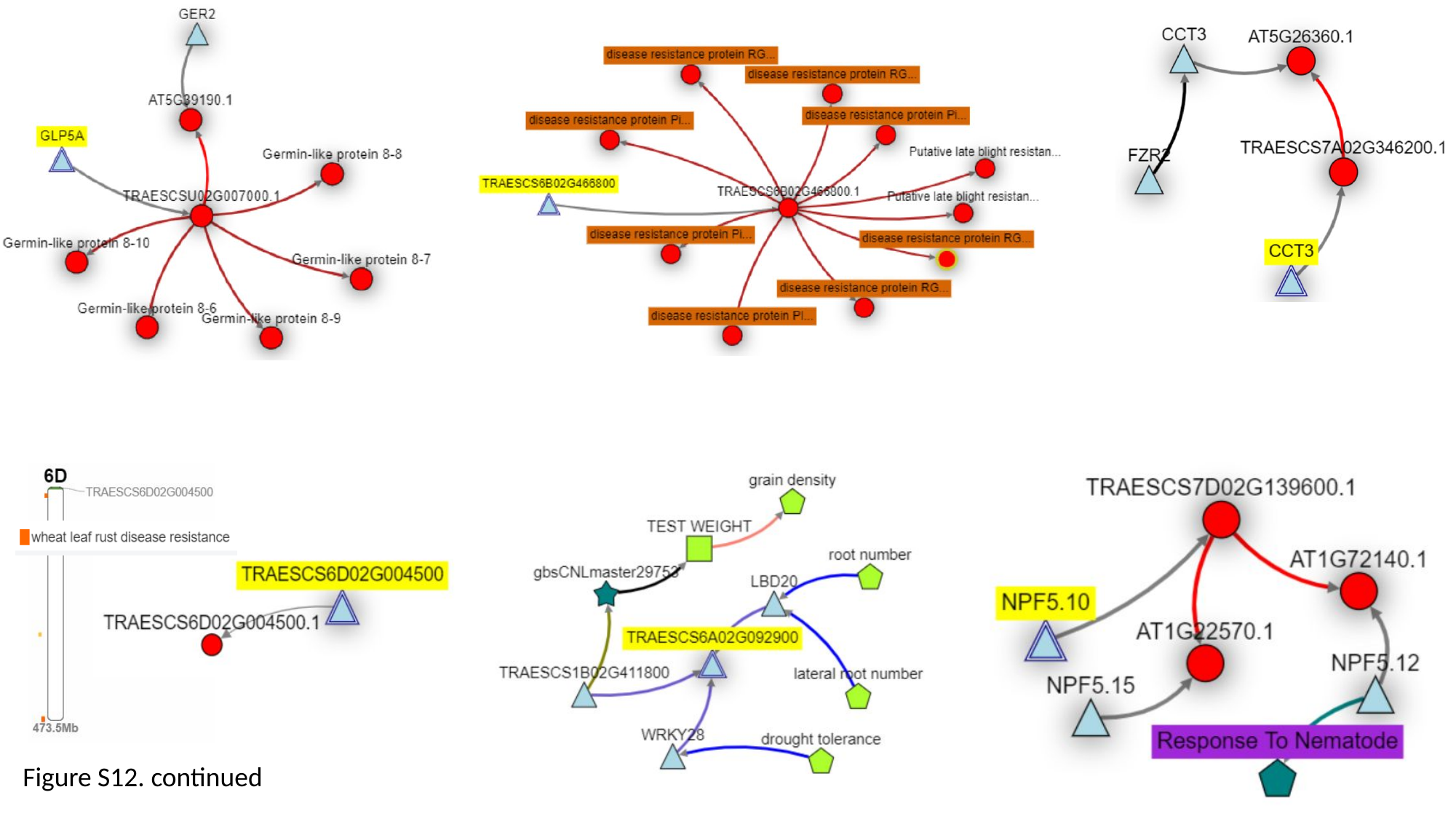

Figure S12. continued

## Slide 22
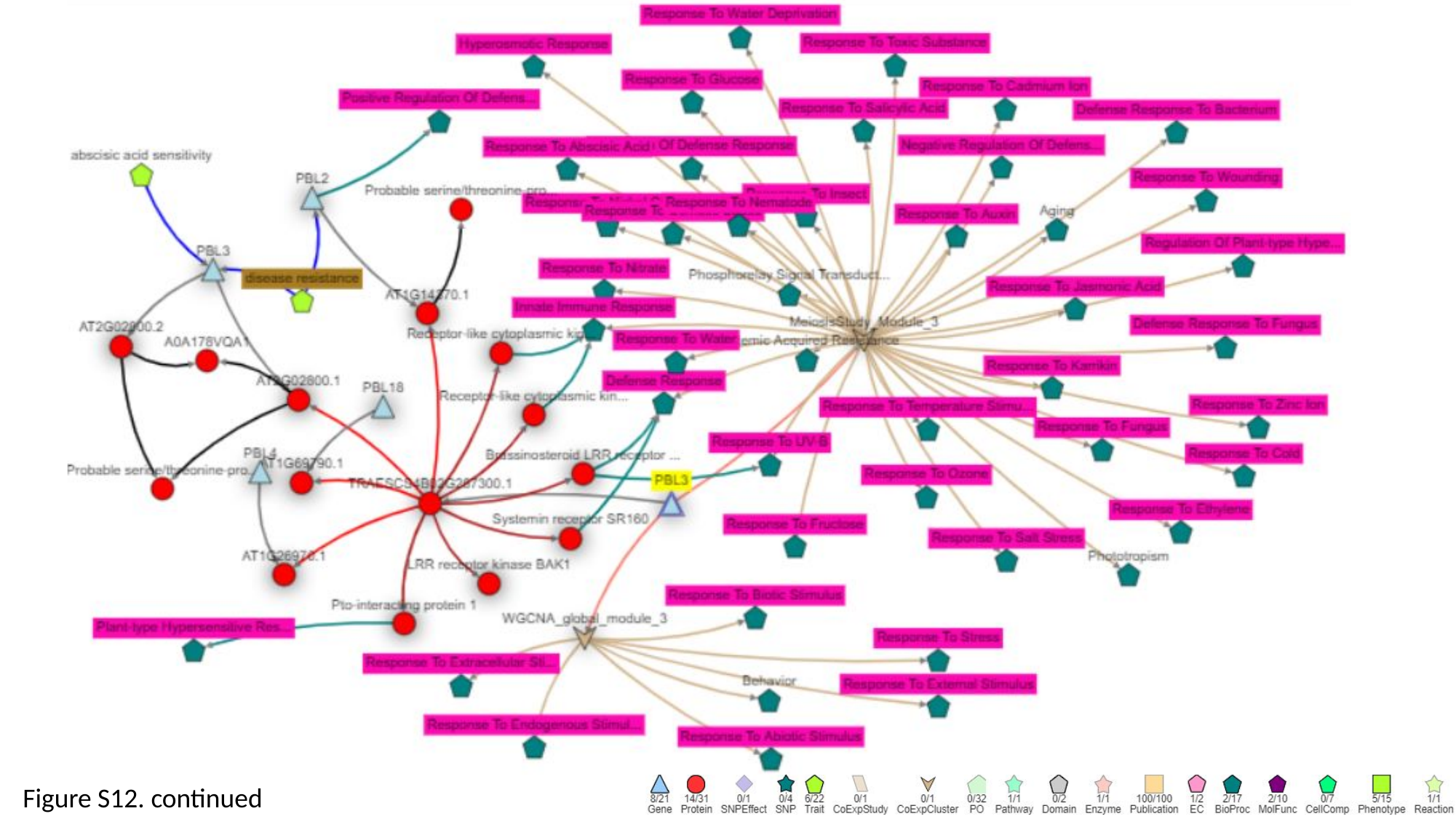

Figure S12. continued

## Slide 23
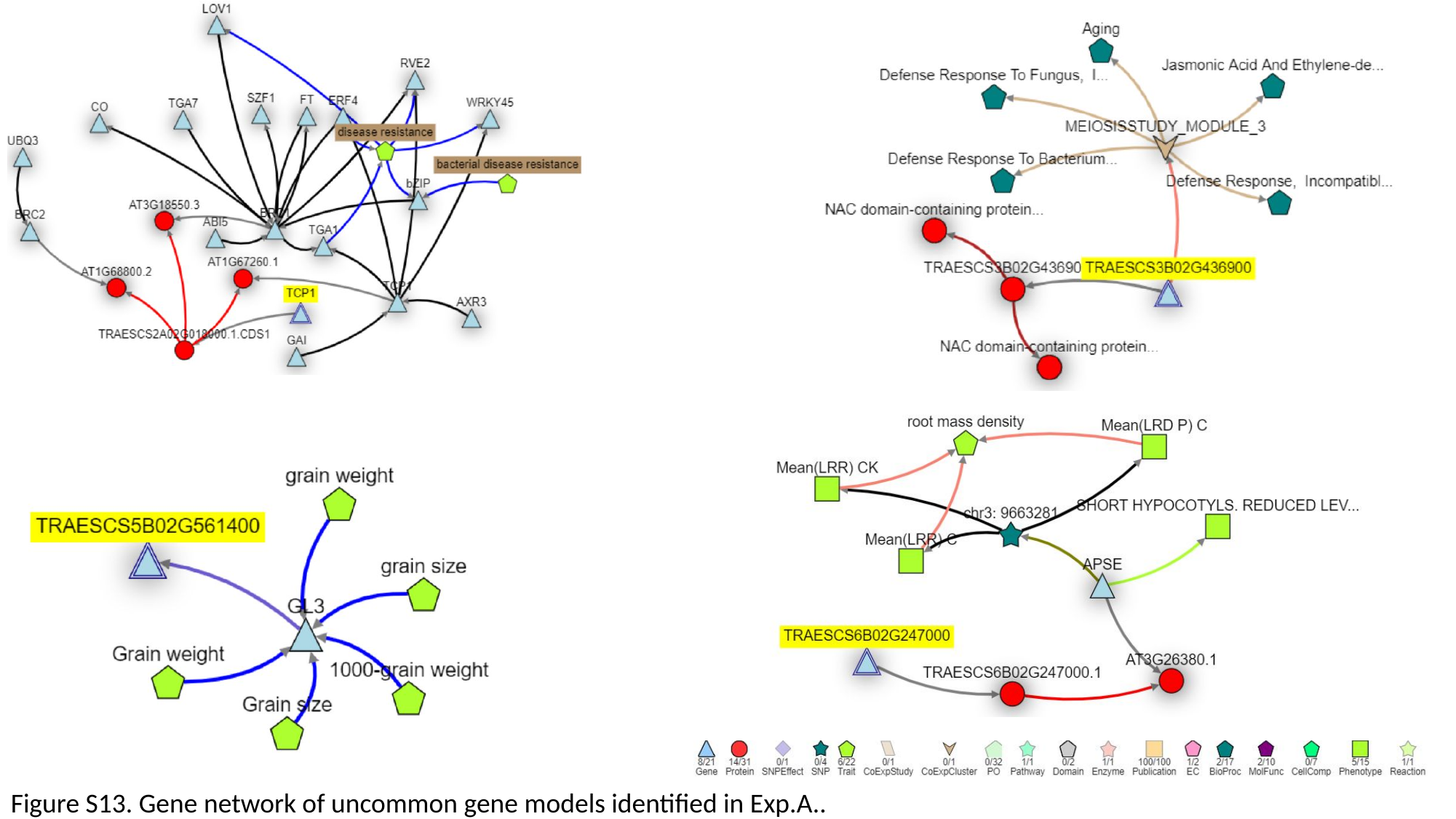

Figure S13. Gene network of uncommon gene models identified in Exp.A..

## Slide 24
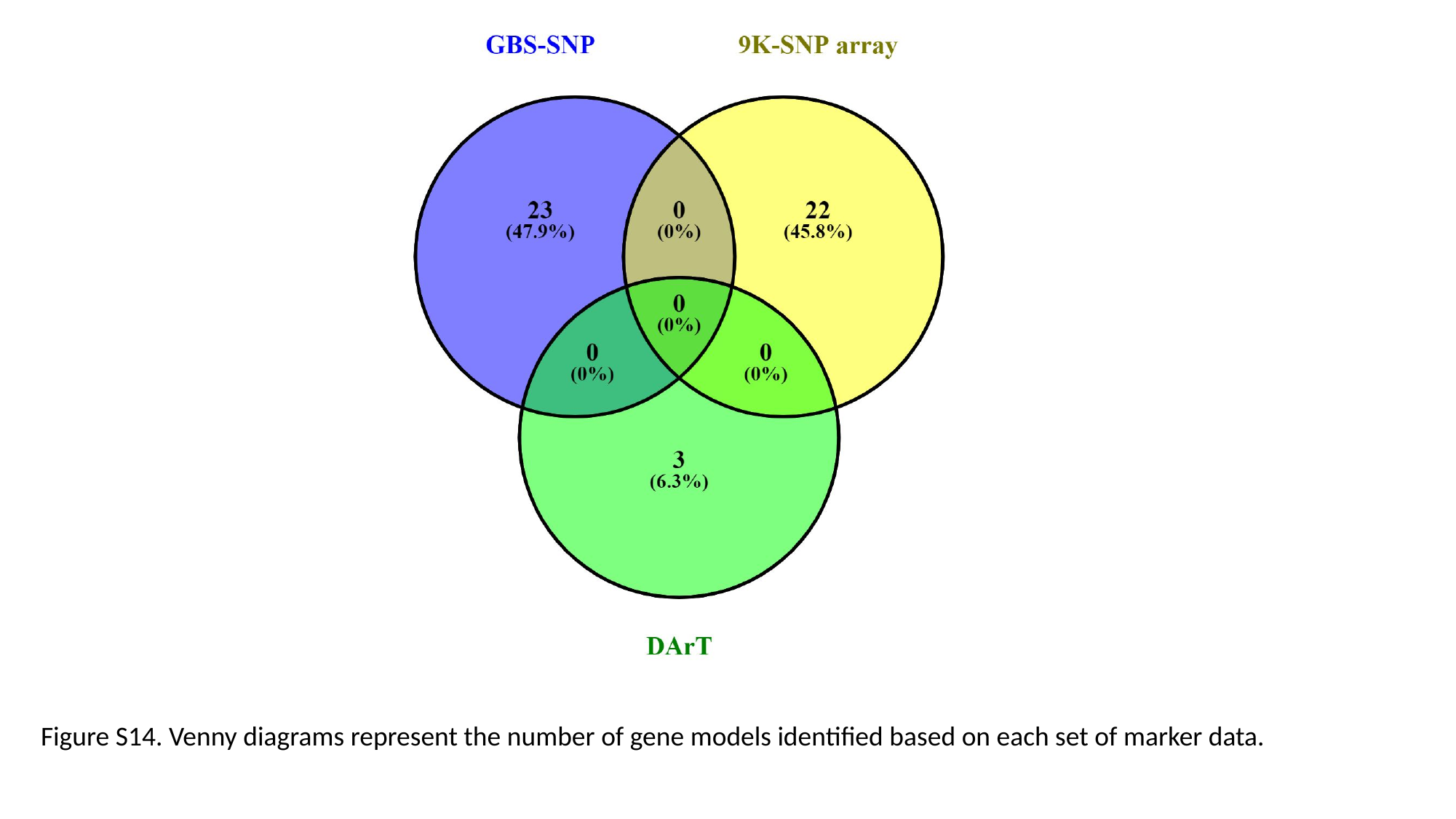

Figure S14. Venny diagrams represent the number of gene models identified based on each set of marker data.
